# Arginine metabolism is a biomarker of red blood cell and human aging

**DOI:** 10.1111/acel.14388

**Published:** 2024-10-30

**Authors:** Julie A. Reisz, Eric J. Earley, Travis Nemkov, Alicia Key, Daniel Stephenson, Gregory R. Keele, Monika Dzieciatkowska, Steven L. Spitalnik, Eldad A. Hod, Steven Kleinman, Nareg H. Roubinian, Mark T. Gladwin, Kirk C. Hansen, Philip J. Norris, Michael P. Busch, James C. Zimring, Gary A. Churchill, Grier P. Page, Angelo D'Alessandro

**Affiliations:** ^1^ Department of Biochemistry and Molecular Genetics University of Colorado Anschutz Medical Campus Aurora Colorado USA; ^2^ RTI International Atlanta Georgia USA; ^3^ Omix Technologies Inc Aurora Colorado USA; ^4^ Department of Pathology and Cell Biology Columbia University Irving Medical Center New York City New York USA; ^5^ University of British Columbia Victoria British Columbia Canada; ^6^ Vitalant Research Institute San Francisco California USA; ^7^ Kaiser Permanente Northern California Division of Research Pleasanton California USA; ^8^ Department of Laboratory Medicine University of California San Francisco San Francisco California USA; ^9^ Department of Medicine University of Maryland School of Medicine, University of Maryland Baltimore Maryland USA; ^10^ Department of Pathology University of Virginia Charlottesville Virginia USA; ^11^ The Jackson Laboratory Bar Harbor Maine USA

**Keywords:** arginine, citrulline, omics, organismal aging, quantitative trait loci, red blood cell metabolism

## Abstract

Increasing global life expectancy motivates investigations of molecular mechanisms of aging and age‐related diseases. This study examines age‐associated changes in red blood cells (RBCs), the most numerous host cell in humans. Four cohorts, including healthy individuals and patients with sickle cell disease, were analyzed to define age‐dependent changes in RBC metabolism. Over 15,700 specimens from 13,757 humans were examined, a major expansion over previous studies of RBCs in aging. Multi‐omics approaches identified chronological age‐related alterations in the arginine pathway with increased arginine utilization in RBCs from older individuals. These changes were consistent across healthy and sickle cell disease cohorts and were influenced by genetic variation, sex, and body mass index. Integrating multi‐omics data and metabolite quantitative trait loci (mQTL) in humans and 525 diversity outbred mice functionally linked metabolism of arginine during RBC storage to increased vesiculation—a hallmark of RBC aging—and lower post‐transfusion hemoglobin increments. Thus, arginine metabolism is a biomarker of RBC and organismal aging, suggesting potential new targets for addressing sequelae of aging.

## Graphical abstract

Reisz et al. examined age‐related changes in erythrocyte metabolism using 15,700 specimens from 13,757 individuals across four cohorts, including healthy donors and sickle cell disease patients. The study identifies alterations in arginine metabolism associated with chronological age, linking them to erythrocyte function, vesiculation, and post‐transfusion outcomes. These changes are consistent across populations and are influenced by genetics (especially polymorphisms in ARG1 and SMOX—validated in 525 diversity outbred mice), sex, and BMI, with implications for transfusion medicine and aging‐related conditions.

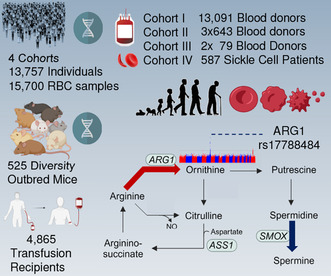

**Abbreviations**
AFR: African American
AFRAMRCN: African American
ARG1: arginase 1
ASN: Asian
ASS1: argininosuccinate synthetase 1
BMI: body mass index
CAU: Caucasian
CPS1: carbamoyl phosphate synthetase I
DIDS: donor iron deficiency study
EAS: East Asian
FDR: false discovery rate
G6PD: glucose‐6‐phosphate dehydrogenase
GLRX: glutaredoxin
HISP: hispanic
HISP1: Mexican and Central American Hispanic
HK1: hexokinase 1
HSP7C: heat shock protein 7C
LDA: linear discriminant analysis
mQTL: metabolite quantitative trait loci
MS: mass spectrometry
NAD: nicotinamide adenine dinucleotide
NADPH: nicotinamide adenine dinucleotide phosphate, reduced
NO: nitric oxide
NOS: nitric oxide synthase
ODC: ornithine decarboxylase
OTC: ornithine transcarbamylase
QTL: quantitative trait loci
RBC: red blood cells
REDS: recipient epidemiology and donor evaluation study
SAS: South Asian
SMOX: spermine oxidase
SNARE: soluble NSF attachment protein receptor
SNP: single nucleotide polymorphism
TGFβ: transforming growth factor beta

## 1 INTRODUCTION

As of 2023, the global average life expectancy is approximately 73.4 years (Feraldi & Zarulli, 2022). In the United States, the aging baby boomer generation (birth years 1946–1964) now comprises ~16% of all Americans according to the 2020 census. Furthermore, current trajectories indicate that, by 2030, 80% of the world's population over 65 will live in low‐ and middle‐income countries (Economic & Affairs, 2020). As longevity increases, so do age‐related comorbidities, including cardiovascular and neurodegenerative diseases (Reisz, Barrett, et al., 2018), resulting in increased socioeconomic burdens requiring updated approaches for addressing looming healthcare crises. Thus, defining the molecular phenotypes of organismal aging is imperative.

Beyond lifespan extension, recent efforts in aging research aim to extend health span (i.e., quality of years lived) (Crimmins, 2015). The ultimate goal is to understand both shared and unique drivers of age‐associated diseases, to then identify medical (e.g., pharmaceuticals, transfusions, and transplantation), dietary, and lifestyle interventions. To this end, studies have addressed mechanisms underpinning age‐related chronic inflammation and oxidant stress, focusing on the interface between the gut/microbiome axis and immunity (Bosco & Noti, 2021; Conway, & N, A. D., 2021).

Omics profiling enables system‐level molecular characterizations of aging in mammals to investigate changes in easy‐to‐access biofluids (e.g., serum or urine), and associate them with age‐related decline in physical function, cognitive ability, and disease. Circulating red blood cells (RBCs) provide a simplified, easily obtained, cell‐centric window into systems metabolism as the most numerous cell type in blood and the human body (Sender et al., 2016). While simple per se, lacking nuclei and organelles, RBCs are a rich matrix for small molecules; indeed, RBCs express at least 77 transporters (Bordbar et al., 2011) and counting (Haiman, D'Alessandro, & Palsson, 2024) that influx and efflux metabolites during exposure to peripheral organs and tissues during 60‐s circuits through the body, from lungs to peripheral capillaries and back, for their ~120 day lifespan. Ultimately, RBC metabolic profiles reflect age‐related changes to erythropoiesis (Filippi & Ghaffari, 2019) and systemic exposures (e.g., diet, habits, and medications) (Nemkov et al., 2020). As such, the “chemical individuality” (Childs, 1970) of blood metabolism informs on remote organ health relative to host biology (e.g., sex, age, and genetics), a founding principle of modern‐day clinical chemistry.

Lacking organelles, RBCs cannot adapt to changing biological conditions during cellular and organismal aging by de novo protein synthesis, damage repair mechanisms, autophagy, and mitochondrial ATP generation, dysregulation of all of which are hallmarks of aging (López‐Otín et al., 2023). Rather, RBCs primarily transport and deliver gases (O_2_ and CO_2_), processes tightly regulated by an intricate metabolic network to maintain energy/redox metabolism as a function of hypoxia, and oxidant stress as RBCs traverse from ~40 mmHg pO_2_ in hypoxic peripheral capillaries to 95–100 mmHg pO_2_ in the lung, a circuit navigated ~172,800 times during their lifespan (Nemkov et al., 2018). Understanding RBC aging in the context of organismal aging is essential for systems physiology and has translational implications. Ex vivo storage of RBCs in blood banks provides ~100 million units of packed RBCs for global transfusions annually, the most common in‐hospital medical intervention after vaccination. Refrigerated RBCs age more rapidly in the blood banks, with a maximal shelf‐life of 42 days. Similar to RBC aging in vivo (D'Alessandro, Anastasiadi, et al., 2023), long‐stored RBCs have dysregulated metabolism, increased oxidant stress, and decreased surface area‐to‐volume ratios due to vesiculation, similar to cellular senescence in vivo (Yoshida et al., 2019). Thus, refrigerator‐stored RBCs in vitro model oxidant‐induced stress responses in vivo, including exercise, hemorrhagic shock, viral infection, and high‐altitude conditions (D'Alessandro, Anastasiadi, et al., 2023). Refrigerator storage‐damaged RBCs also recapitulate organismal aging‐associated changes in RBC morphology and splenic sequestration (Roussel et al., 2021).

RBCs also provide systemic‐level information for aging research. RBC mean corpuscular volumes (Brzezniakiewicz‐Janus et al., 2020; Gamaldo et al., 2011; Lee et al., 2022) and distribution widths are among the best current predictors of all‐cause morbidity and mortality (Pan et al., 2019; Patel et al., 2009; Perlstein et al., 2009). As such, it has been proposed that RBCs may provide a simplified model of cellular aging (Kaestner & Minetti, 2017). Nonetheless, RBC metabolism has yet to be characterized in large human cohorts, the focus of the present study, which explored metabolic correlates of human chronological age in >13,700 humans and 525 diversity outbred mice. Using multi‐omics approaches and genome‐wide association studies in humans and mice, we identified a heretofore unrecognized role for age‐related changes in arginine metabolism in the context of RBC and organismal aging.

## 2 METHODS

### 2.1 REDS index cohort

Volunteer enrollment into the Recipient Epidemiology and Donor evaluation Study (REDS) RBC‐Omics (https://biolincc.nhlbi.nih.gov/studies/reds_iii/) was described previously (Endres‐Dighe et al., 2019). Units from 13,091 donors were leukocyte‐filtered, stored for 42 days under standard refrigerated conditions in either Additive Solution (AS)‐1 or AS‐3, and tested for hemolytic parameters as described (D'Alessandro, Culp‐Hill, et al., 2019; Kanias et al., 2017; Lanteri et al., 2019). Packed RBC samples (*n* = 13,091) were analyzed by mass spectrometry‐based metabolomics as described (Nemkov, Key, et al., 2024; Nemkov, Stephenson, et al., 2024).

### 2.2 REDS recall cohort

Index donors whose end‐of‐storage (42 d) units exhibited the highest and lowest hemolysis measurements (<5th and >95th percentile) were recalled for a second donation. The second unit (*n* = 643) was tested at storage Days 10, 23, and 42 for hemolytic parameters and mass spectrometry‐based metabolomics (Nemkov et al., 2022) and proteomics (Reisz, Nemkov, et al., 2018) within the REDS‐IV‐P study (*n* = 1929 samples analyzed) (Josephson et al., 2022).

### 2.3 Metabolite quantitative trait loci analysis of index donors

Metabolite quantitative trait loci (mQTL) analyses of arginine metabolites were performed as described for kynurenine (Nemkov, Stephenson, Erickson, et al., 2024), acylcarnitines (Nemkov, Key, Stephenson, et al., 2024), and glycolytic metabolites (Nemkov, Stephenson, et al., 2024) in the REDS index cohort. Details of the genotyping and imputation of RBC Omics study participants were previously described (Guo, Busch, Seielstad, Endres‐Dighe, Westhoff, Keating, Hoppe, Bordbar, Custer, Butterworth, Kanias, Mast, Kleinman, Lu, & Page, 2019; Page et al., 2021). Genotyping was performed using a Transfusion Medicine microarray (879,000 single‐nucleotide polymorphisms—SNPs); data were deposited in dbGAP accession number phs001955.v1.p1. Imputation was performed using 811,782 SNPs that passed quality control. After phasing using Shape‐IT (Delaneau et al., 2008), imputation was performed using Impute2 (Howie et al., 2011) with the 1000 Genomes Project phase 3 (Howie et al., 2011) all‐ancestry reference haplotypes. R package SNPRelate (Zheng et al., 2012) was used to calculate ancestry principal components (PCs). Association analyses for arginine pathway metabolites used an additive SNP model in the R package ProbABEL (Aulchenko et al., 2010) on the 13,091 study participants who had both metabolomics data and imputed genotype data on serial samples from stored RBC components that passed respective quality control procedures. We adjusted for sex, age (continuous), blood donation frequency in the last 2 years (continuous), blood donor center, and 10 ancestry PCs. Statistical significance was determined using a FDR‐corrected p‐value threshold of 5 × 10^−8^. We only considered variants with a minimum minor allele frequency of 1% and a minimum imputation quality score of 0.80. OASIS (Omics Analysis, Search & Information, a TOPMED funded resource (Perry et al., 2021)) was used to annotate the top SNPs. OASIS annotation includes information on position, chromosome, allele frequencies, closest gene, type of variant, position relative to closest gene model, predicted functional consequences, tissue‐specific gene expression, and other information.

### 2.4 Donor iron deficiency study

A total of 79 repeat blood donors were enrolled as part of the donor iron deficiency study (DIDS) study for baseline and post‐iron repletion, as described (Hod et al., 2022). For each donor, two samples were available for metabolomics analyses, totaling 158 samples.

### 2.5 Walk‐PHaSST

SCD patients were recruited for the Treatment of Pulmonary Hypertension and Sickle cell disease with Sildenafil Therapy (Walk‐PHaSST) study screening phase. A total of 587 SCD patients with available RBC samples were used for metabolomics analyses. Eligibility and exclusion criteria were described previously (D'Alessandro, Anastasiadi, Tzounakas, Nemkov, Reisz, Kriebardis, Zimring, Spitalnik, & Busch, 2023; D'Alessandro, Nouraie, Zhang, Cendali, Gamboni, Reisz, Zhang, Bartsch, Galbraith, Espinosa, et al., 2023).

### 2.6 REDS vein‐to‐vein database

We interrogated the BioLINCC database to access public use data from the NHLBI REDS cohort as previously described (“https://biolincc.nhlbi.nih.gov/studies/reds_iii/”; Karafin et al., 2017; Roubinian et al., 2019; Roubinian et al., 2022) and discussed below. The database includes blood donor, component manufacturing, and patient data from 12 academic and community hospitals from four geographically diverse regions in the United States from January 1, 2013 to December 31, 2016. Arginine and citrulline levels from the REDS Index donors (Endres‐Dighe et al., 2019a) were linked to hemoglobin increments in adult patients receiving one RBC unit during one or more transfusion episodes in the 4‐year timeframe. Hemoglobin increments are defined as a change in hemoglobin (ΔHb; g/dL) following a single RBC unit transfusion episode, calculated as the difference between post‐transfusion and pre‐transfusion levels, adjusted for donor age, sex, hemoglobin, component apheresis, irradiation, storage age, storage solution, recipient age, sex, body mass index, and hemoglobin level. Pre‐transfusion hemoglobin was the most proximal hemoglobin measurement prior to transfusion, at most 24 h prior. Post‐transfusion hemoglobin was defined as the laboratory measure nearest to 24 h post‐transfusion (between 12 and 36 h). Association of the levels of arginine and citrulline: arginine ratios in end‐of‐storage units from the same donor were correlated by tertiles to changes in post‐transfusion hemoglobin increments. Two‐sided p‐values <0.05 were considered statistically significant. Analyses were performed using Stata Version 14.1, StataCorp, College Station, TX.

### 2.7 Diversity outbred mouse cohort

This diversity outbred (DO) cohort of 525 mice have previously been described (Nemkov, Key, Stephenson, et al., 2024; Nemkov, Stephenson, et al., 2024). The DO population was produced through extensive outbreeding (>50 generations) of eight genetically distinct inbred strains: A/J, C57BL/6J, 129S1/SvlmJ, NOD/ShiLtJ, NZO/HILtJ, CAST/EiJ, PWK/PhJ, and WSB/EiJ. Mice were obtained in six batches (ranging from 77 to 95 animals), each of a single sex. Cumulatively, the cohort consists of 263 females and 262 males. All animals were genotyped using the GigaMUGA array (143,259 SNPs) (Morgan & Welsh, 2015).

### 2.8 Mouse RBC storage

RBCs were collected from each animal and stored for 7 days as described previously (Howie et al., 2019). Mass spectrometry metabolomics profiling was performed on fresh and stored samples from each mouse in 96‐well plate format on a Thermo Vanquish UHPLC coupled to a Thermo Q Exactive mass spectrometer using a 1‐min ultra‐high‐throughput C18 gradient as described (Nemkov, Key, Stephenson, et al., 2024). Seven stored samples were identified as outliers by principal component analysis and removed from further analysis.

### 2.9 mQTL analysis of DO mice

The mQTL analysis for this DO cohort uses the qtl2 R package (Broman et al., 2019), which has been used for this cohort previously (Nemkov, Key, Stephenson, et al., 2024; Nemkov, Stephenson, et al., 2024). Briefly, zeros in the metabolite data were converted to missing values and metabolites with fewer than 100 non‐missing observations were filtered out from further analyses. Each metabolite variable was transformed to normal quantiles to mitigate the effects of outliers which can result in false‐positive mQTL. The mQTL analysis was performed based on founder strain haplotypes probabilistically reconstructed at genome‐wide loci (137,192 SNPs), which allows the QTL model to characterize the genetic effect in terms of eight alleles. Sex and mouse batch were adjusted for as covariates. A linear mixed effect model (LMM) was used, with a random effect that accounts for overall relatedness (i.e., kinship). A lenient significance threshold of LOD score >6 was used to allow more comparisons between mouse and human mQTL; for reference, a LOD score >8 approximately represents a genome‐wide significant QTL (Keele, 2023). SNP association was performed at detected mQTL of interest by imputing variant genotypes based on the distribution of SNP alleles across the founder strains (GRCm39). The same analysis framework was used for both fresh and stored samples separately.

### 2.10 Sex difference analysis of DO mice

Differences in metabolite levels between the sexes were tested for using a LMM fit with the lme4 [10.18637/jss.v067.i01] and lmerTest [10.18637/jss.v082.i13] R packages. A per metabolite p‐value was obtained using ANOVA to compare a model that included sex as a covariate to a null model excluding it, both with mouse batch included as a random effect. Sex differences were detected based on FDR <0.1[https://www.jstor.org/stable/2346101].

### 2.11 Statistical analysis

Correlation analyses (Spearman) were performed using MetaboAnalyst 5.0. (Pang et al., 2021) Correlation plots were generated with R (R version 4.2.3 (2023‐03‐15), https://www.r‐project.org/).

## 3 RESULTS

### 3.1 Arginine pathway metabolites are dysregulated in aging

**FIGURE 1:**
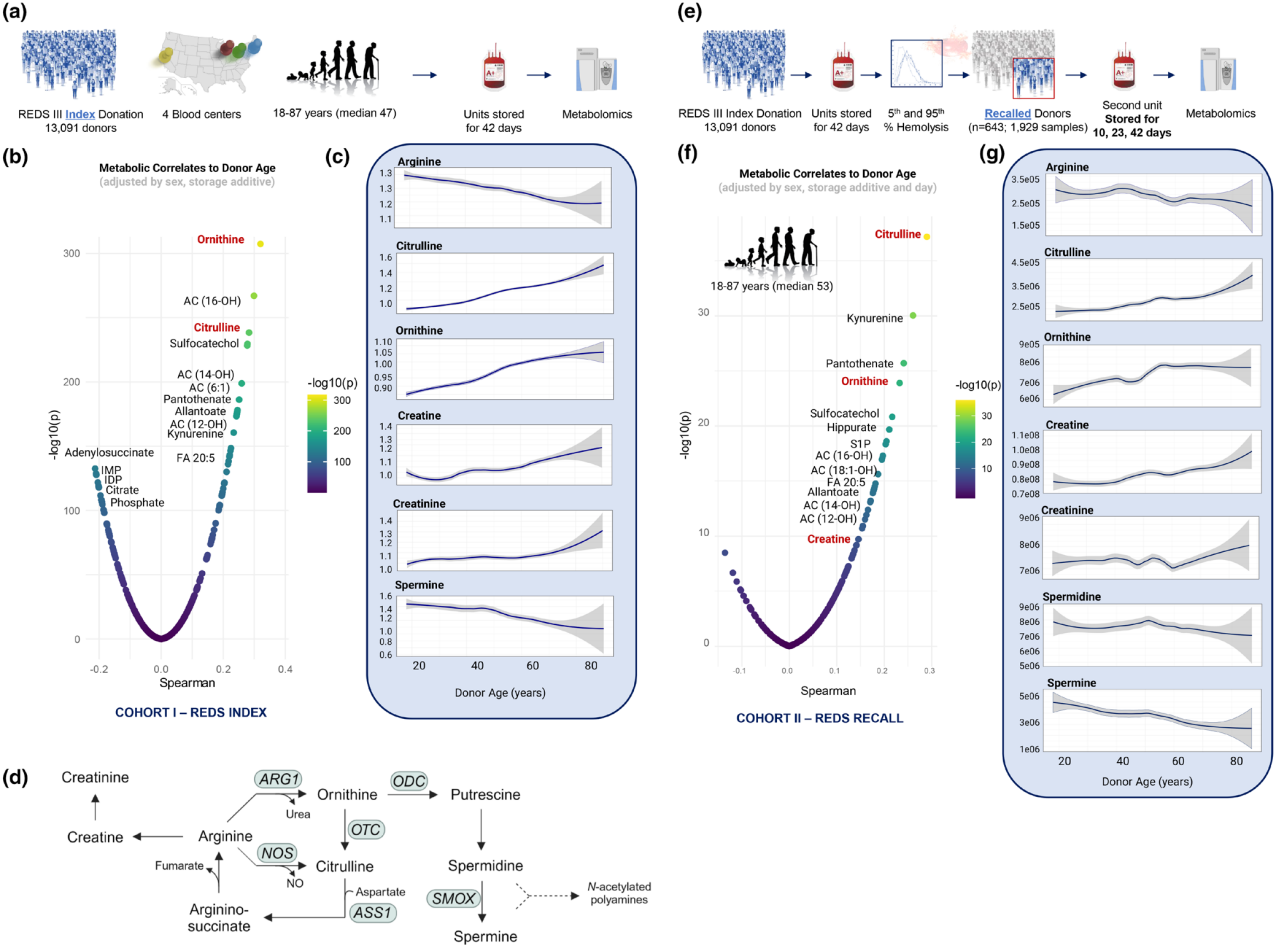
Arginine metabolites are impacted as a function of donor chronological age in red blood cells of healthy donors at 42 days of storage. (a) Overview of the index arm of the REDS study whereby 13,091 healthy individuals donated a unit of blood which was stored for 42 days and characterized by mass spectrometry (MS)‐based metabolomics. (b) Spearman correlation of RBC metabolite levels with donor chronological age following adjustment for sex and storage additive. (c) Relative levels of arginine pathway metabolites as a function of donor chronological age in the index donors. Lines represent median (dark blue) +/− quartile ranges (shaded gray). (d) Overview of arginine metabolism. ARG1, arginase 1; NOS, nitric oxide synthase; ODC, ornithine decarboxylase; OTC, ornithine transcarbamylase; ASS1, argininosuccinate synthetase 1; SMOX, spermine oxidase. (e) Overview of the recall arm of the REDS study in which the donors whose units represented the extremes of hemolytic propensity (at 42 days) donated a second unit which was sampled at Days 10, 23, and 42 of storage followed by MS metabolomics. (f) Spearman correlation of RBC metabolite levels with donor chronological age following adjustments for sex, storage additive, and storage day. (g) Relative levels of arginine pathway metabolites as a function of donor chronological age in the recall units independent of storage duration. Lines represent median (dark blue) +/− quartile ranges (shaded gray).

As part of REDS, a single RBC unit was collected from each of 13,091 healthy human volunteer donors, stored for 42 days (i.e., outdate in the United States), and characterized by high‐throughput mass spectrometry‐based metabolomics (Figure 1a). Spearman correlation of RBC metabolite levels with donor chronological age identified purine metabolites (adenylosuccinate, IMP, IDP, and phosphate) as predominant negative correlates, while arginine metabolism intermediates ornithine and citrulline were two of the top positive correlates to donor chronological age, alongside hydroxylated acylcarnitines, the coenzyme A precursor pantothenate, and kynurenine, the strongest metabolic biomarker of osmotic fragility in stored RBCs (Figure 1b, Table S1) (Nemkov, Stephenson, Erickson, et al., 2024). Additional arginine metabolism pathway intermediates demonstrated donor chronological age dependency as well, including the nitric oxide synthase (NOS) product arginine (0.76 fold change when comparing the oldest 5% of donors to the youngest 5% of donors, *p* = 1.01e‐13), citrulline (1.34 FC, *p* = 7.46e‐53), and the arginase product ornithine (1.23 FC, *p* = 7.27e‐72, Figure 1c,d). Creatine and creatinine levels also increased as a function of donor chronological age (FC 1.08, *p* = 1.54e‐5 and FC 1.06, *p* = 1.16e‐6, respectively) while spermine decreased (FC 0.77, *p* = 1.44e‐8).

In a subsequent phase of REDS, index donors whose first donated units ranked at the extreme 5th or 95th percentile with respect to hemolytic propensity (*n* = 643) were recalled for a second donation; the second unit was sampled at Days 10, 23, and 42 of refrigerated storage (Figure 1e—1929 specimens) (Endres‐Dighe et al., 2019b). Characterizing this cohort, after adjusting for storage additive and RBC storage duration, confirmed citrulline, ornithine, hydroxylated acylcarnitines (e.g., C16‐OH, C14‐OH, and C12‐OH), pantothenate, and kynurenine as top positive correlates of age (Figure 1f, Table S1). Arginine steadily decreased and citrulline increased as a function of donor age (Figure 1g). Donor chronological age 50–55 emerged as an inflection point of the arginine pathway, after which ornithine plateaued, creatine and creatinine sharply increased, and spermidine and spermine decreased. Variability in metabolite levels at later ages in both cohorts can be explained, in part, by fewer elderly donors.

**FIGURE 2:**
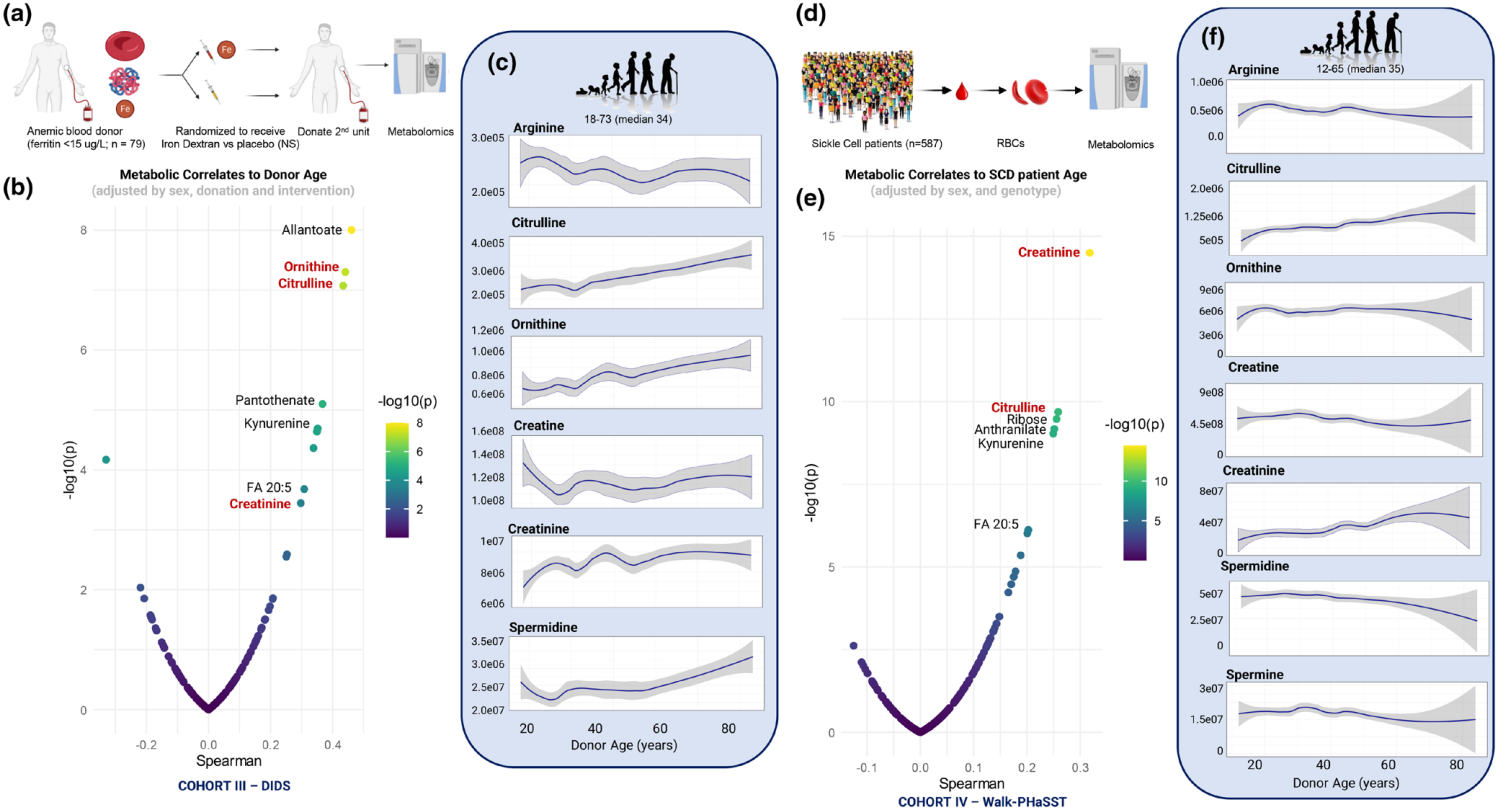
Arginine metabolites are impacted by donor chronological age in red blood cells of donors with anemia or sickle cell disease. (a) Overview of the DIDS study testing whether iron dextran improved the quality of stored units from donors with anemia from repeated donations. Units were sampled before and after the iron dextran course and profiled by mass spectrometry (MS)‐based metabolomics. (b) Spearman correlation of RBC metabolite levels with donor chronological age following adjustment for sex, donation time point, and intervention. (c) Relative levels of arginine pathway metabolites as a function of donor chronological age independent of storage time point. Lines represent median (dark blue) +/− quartile ranges (shaded gray). (d) Overview of the Walk‐PHaSST study of sickle cell disease followed by MS metabolomics. (e) Spearman correlation of RBC metabolite levels with patient chronological age following adjustments for sex and Hb genotype. (f) Relative levels of arginine pathway metabolites as a function of patient chronological age. Lines represent median (dark blue) +/− quartile ranges (shaded gray).

To validate these findings in an independent cohort, we correlated RBC metabolic profiles from leukocyte‐filtered blood units donated by 79 iron‐deficient repeat‐donor volunteers in DIDS, a clinical trial testing whether intravenous iron dextran normalized hematological parameters, RBC storage quality, and post‐transfusion performance (Bitan et al., 2019). This cohort shared a feature commonly observed in aging, as anemia occurs in older individuals contributing significantly to morbidity and mortality (Stauder & Thein, 2014). A baseline unit from each DIDS volunteer was stored for 42 days; volunteers were then randomized to receive either iron dextran or placebo. Four to six months later, they donated a second unit which was stored similarly (Figure 2a). Metabolomics was performed on RBC specimens from both donations (Hod et al., 2022). After adjusting for sex, donation timepoint, and intervention arm, top positive correlates with donor chronological age included ornithine (top correlate overall), citrulline, and creatinine (Figure 2b, Table S1). Several other results recapitulated observations from both REDS cohorts, including allantoate and kynurenine. Consistent with the REDS cohort, arginine levels in DIDS donor RBCs decreased in a chronological age‐dependent manner, while its immediate catabolites citrulline and ornithine increased (Figure 2c). Changes to creatine and creatinine were most evident before age 40, while an increase in spermidine occurred at age >60.

As the REDS and DIDS cohorts included healthy donors, we expanded our investigation to study a cohort of non‐healthy subjects suffering from a common hemoglobinopathy, sickle cell disease (SCD). SCD is associated with shorter lifespan and early onset of cardiorenal, pulmonary, and cognitive dysfunction; indeed some suggest that it represents an accelerated aging syndrome (Idris et al., 2022). Age‐centered bioinformatics elaborations of previously published (D'Alessandro, Nouraie, Zhang, Cendali, Gamboni, Reisz, Zhang, Bartsch, Galbraith, Gordeuk, & Gladwin, 2023) data from the Walk‐PHaSST trial (*n* = 587 patients, Figure 2d) identified RBC creatinine, citrulline, and tryptophan metabolites (anthranilate and kynurenine) as top positive correlates of age, after adjusting for sex and hemoglobin S/C genotype (Figure 2e, Table S1). Notably, Walk‐PHaSST enrolled younger subjects with a median age of 35 years, compared with REDS and DIDS, which relied on healthy volunteer blood donors. Consistent with DIDS, RBCs from SCD patients who required transfusions throughout their lifespan (when HbA <20% (D'Alessandro, Anastasiadi, Tzounakas, Nemkov, Reisz, Kriebardis, Zimring, Spitalnik, & Busch, 2023)) demonstrated linear increases in citrulline with patient chronological age. For other arginine metabolites, levels remained steady across until middle age, followed by age‐dependent increases in creatinine and decreases in arginine, spermidine, and spermine (Figure 2f). In contrast to healthy donor cohorts, SCD RBC ornithine levels did not substantially change with age, perhaps because of a potential cardiovascular survival benefit of increased arginine bioavailability for nitric oxide‐dependent NO and citrulline synthesis, rather than arginase activity in this population (D'Alessandro, Anastasiadi, Tzounakas, Nemkov, Reisz, Kriebardis, Zimring, Spitalnik, & Busch, 2023; Morris et al., 2005). In addition, creatinine:creatine ratios, a hallmark of renal dysfunction (D'Alessandro, Nouraie, Zhang, Cendali, Gamboni, Reisz, Zhang, Bartsch, Galbraith, Espinosa, et al., 2023) that tend to increase with aging, changed direction beyond age 60.

### 3.2 Effects of donor demographics on arginine pathway metabolites

**FIGURE 3:**
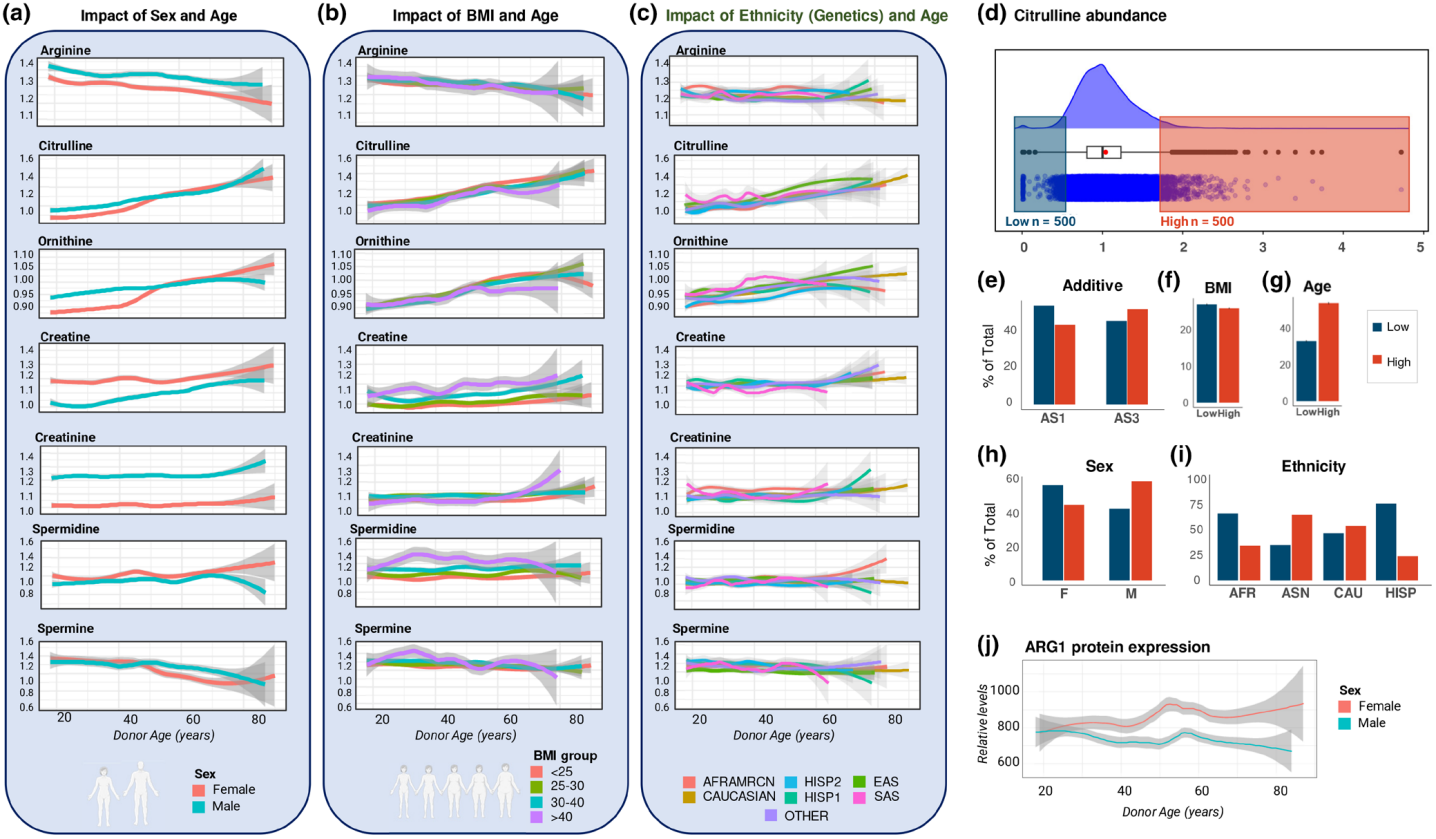
Donor biology impacts age‐related changes to RBC arginine metabolism. (a) Relative levels of arginine and its downstream metabolites in the REDS index cohort separated by sex. (b) Relative levels of arginine and its downstream metabolites in the REDS index cohort as a function of donor BMI. (c) Relative levels of arginine and its downstream metabolites in the REDS index cohort as a function of donor ethnicity. AFRAMRCN, African American; HISP1, Mexican and Central American Hispanic; HISP2, Caribbean Island Hispanic; EAS, East Asian; SAS, South Asian; OTHER, donors who self‐identified as Native Americans, Native Hawaiians, Native Alaskans, multiple races, or were from countries sparsely represented in this cohort such as Iran and the Philippines. (d) Raincloud plot of RBC citrulline levels highlighting the top and bottom 500 donors. (e–i) Representation among the donors with top and bottom 500 end‐of‐storage citrulline levels across storage additive (e), BMI (f), chronological age (g), sex (h), and ethnicity (i), each shown as a percentage of total for the 1000 donor subset identified in (d). AFR, African American; ASN, Asian; CAU, Caucasian; HISP, Hispanic. Data are presented as median +/− SEM. (j) Arginase 1 (ARG1) protein expression in RBCs in the REDS recall cohort as a function of sex and donor chronological age via mass spectrometry‐based proteomics.

After confirming an association of arginine metabolism with the chronological age of the subject in four cohorts, we further dissected metabolomics data for the largest one (REDS Index, *n* = 13,091) in relation to other biological factors, including sex and body mass index (BMI). Sex dimorphism was evident in multiple arginine pathway metabolites (Figure 3a). RBCs from females had lower arginine and creatinine, and higher creatinine and spermidine, across the donor chronological age spectrum (Figure 3a). Interestingly, the sex dimorphism for citrulline, ornithine, and spermine disappears at age 50–52, the average age of menopause onset in the United States (Gold, 2011). Donor age‐associated changes in RBC arginine, citrulline, ornithine, and creatinine were largely BMI‐independent, except for older, high BMI donors (purple line), with decreased ornithine and citrulline and increased creatinine (Figure 3b, Table S1). Creatine and spermidine positively correlated with BMI over the donor age spectrum.

The genetic underpinnings driving chronological age‐related alterations of arginine metabolism were first evaluated using donor‐provided ethnicity information. Ethnicity‐related phenotypic metabolite differences were observed, particularly higher citrulline and ornithine in young South Asian (SAS, pink line) and East Asian (EAS, green) donors above age 40 (Figure 3c **)**. In addition, RBC creatine was lower in SAS donors at nearly all ages. To characterize other demographics that contributed most to altered RBC arginine metabolism, we focused on the top and bottom 500 index donors for levels of citrulline (NOS product, Figure 3d), ornithine (Figure S1a), and arginine (Figure S1b). Storage additive contributed only minimally to differences in citrulline levels, demonstrating donor‐intrinsic features of arginine metabolism (Figure 3e,f). BMI had no appreciable impact; the variance between the two subgroups was driven more by age (Figure 3g), sex (Figure 3h), and genetic ancestry (Figure 3i). In particular, low RBC citrulline was most likely to be detected in donors of African and Hispanic descent, while donors of Asian descent were over‐represented in the high‐citrulline group. Proteomics data from the REDS recall cohort (*n* = 643 donors, 1929 specimens) revealed a sex dimorphism with respect to arginase 1 (ARG1), an abundant RBC enzyme (D'Alessandro, Reisz, et al., 2019) that converts arginine to ornithine. RBCs from female donors displayed consistently higher ARG1 levels and an age‐dependent increase above age 60, whereas expression decreased in older males (Figure 3j). Though NOS data were not available in our proteome profile, perhaps due to the trace levels in mature RBCs (Leo et al., 2021), the sex‐dependent ARG1 trend is consistent with lower citrulline in females (Figure 3a,h), suggesting that the ARG1/ornithine route is favored in female RBCs in contrast to males.

### 3.3 Genetic basis for altered arginine metabolism in stored human RBCs

**FIGURE 4:**
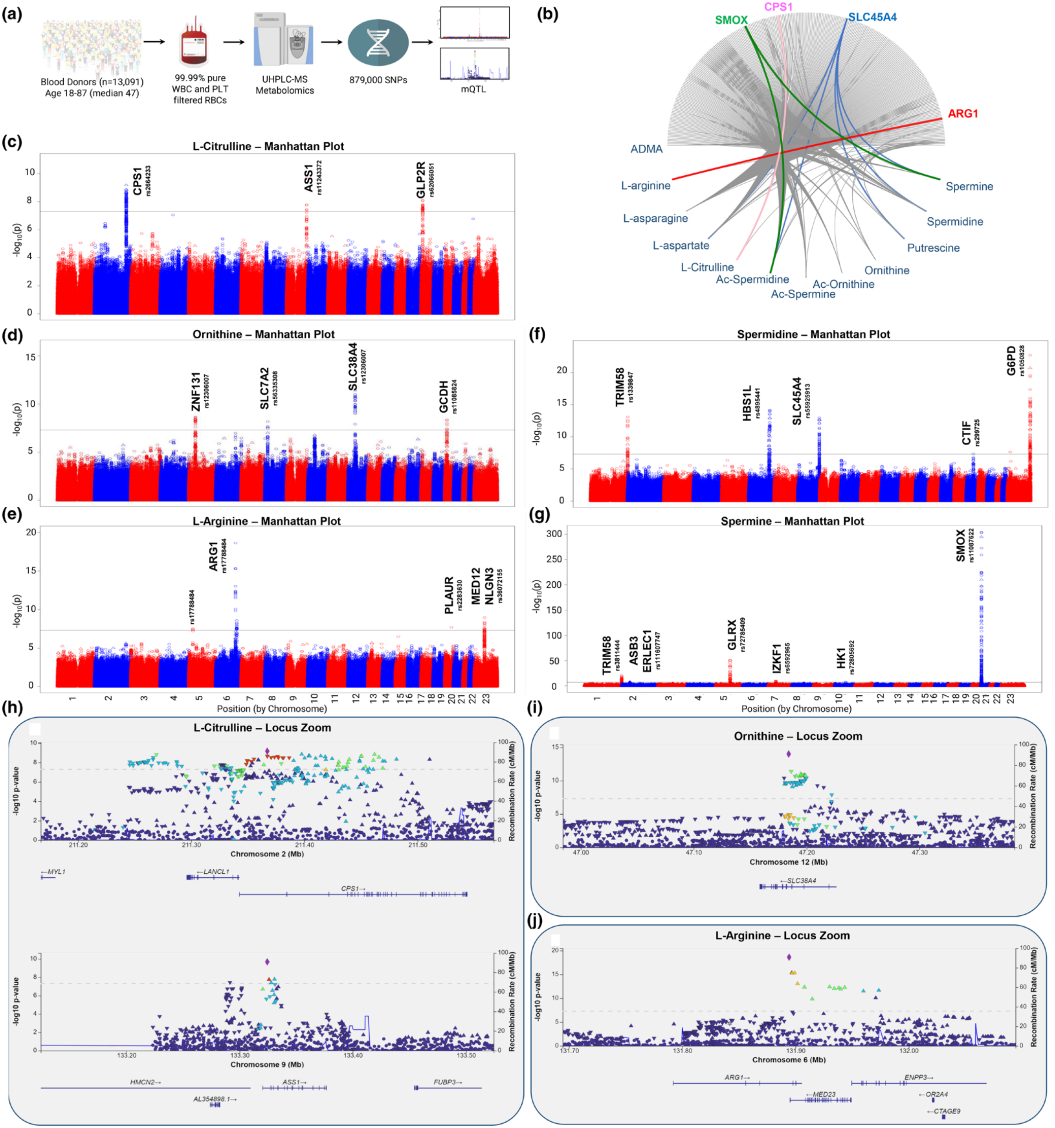
Metabolite Quantitative Trait Loci (mQTL) analysis identifies genetic underpinnings associated with RBC arginine metabolism. (a) End‐of‐storage units from REDS index donors (*n* = 13,091) were profiled using a precision transfusion medicine array to monitor the frequency of 879,000 known single nucleotide polymorphisms which were correlated to metabolomics data using mQTL. (b) Circos plot highlighting key SNP–metabolite associations in the arginine pathway. (c–g) Manhattan plots of all significant hits (FDR *p*‐value < 5 × 10^−8^, black horizontal lines), including metabolite‐gene pairs. Each data point corresponds to a –log10(*p*) from a multivariant linear regression model *p*‐value for a given SNP. Manhattan plots are shown for (c) citrulline, (d) ornithine, (e) arginine, (f) spermidine, and (g) spermine. Selected locus zoom plots identify nearest coding regions to which identified SNPs map for (h) citrulline on chromosomes 2 (top) and 9 (bottom), (i) ornithine on chromosome 12, and (j) arginine on chromosome 6.

Because of the apparent effect of genetic factors (e.g., sex dimorphism and ancestry) on end‐of‐storage arginine metabolite levels, we further investigated genetic underpinnings associated with this heterogeneity. For the 13,091 donors in the REDS index cohort, a precision transfusion medicine array (Guo, Busch, Seielstad, Endres‐Dighe, Westhoff, Keating, Hoppe, Bordbar, Custer, Butterworth, Kanias, Mast, Kleinman, Lu, & Page, 2019) evaluated 879,000 SNPs. Using arginine metabolites as Quantitative Trait Loci (mQTL—Figure 4a), multiple genetic regions were significantly associated to cross‐donor variance in arginine metabolism; these are summarized using circos plots linking arginine pathway metabolites to the closest products of the nearest genetic coding region to which they mapped by the mQTL analysis (e.g., the solute carrier SLC45A4 with multiple polyamines) (Figure 4b). Highly significant associations (−log10(p) ranging from 20 to 300) were observed for multiple metabolites and genetic regions coding for well‐established rate‐limiting enzymes in cationic amino acid transport and metabolism (Table S1). For example, the top SNP associations one of the strongest correlates to chronological age, citrulline, mapped to coding regions for rate‐limiting enzymes of the urea cycle: carbamoyl phosphate synthetase 1 (CPS1) (Figure 4c, LocusZoom—previously associated with persistent pulmonary hypertension (Kaluarachchi et al., 2018), a common comorbidity in SCD) and argininosuccinate synthetase (ASS1) (Figure 4h), the latter part of the citrulline‐NO cycle. Another top association with citrulline was glucagon‐like peptide 2 receptor (GLP2R, missense mutation rs17681684—Figure S2a). Top SNPs for ornithine (Figure 4d) were in genes encoding the solute carriers SLC38A4 (Figure 4i)—a cationic and neutral amino acid transporter—and SLC7A2—another cationic amino acid transporter that also carries polyamines—along with zinc finger protein ZNF131 and glutaryl‐CoA dehydrogenase (GCDH), the latter implicated in glutaric aciduria upon dysfunction of cationic (lysine, hydroxylysine) amino acid catabolism (Figure S2b).

Arginine levels were associated with a SNP in the region encoding arginase 1 (ARG1), which converts arginine to ornithine (Figure 4e,j). Spermidine levels were most significantly associated with a SNP on glucose 6‐phosphate dehydrogenase (G6PD, Figure S2c), the chromosome X encoded rate‐limiting enzyme of the pentose phosphate pathway, the main source of NADPH generation in mature RBCs. G6PD mutations are observed in ~500 million individuals worldwide, representing the most common human enzymopathy (Luzzatto et al., 2020) (Figure 4f). Levels of putrescine and *N*‐acetylated polyamines, a modification preventing polyamine export from cells, were also associated with G6PD SNPs (Figure S3a–c). Other polyamine‐associated SNPs were identified on TRIM58, a regulator of late erythropoiesis (Thom et al., 2014) (*N*‐acetylspermidine—Figure S4a, putrescine—Figure S1c, spermidine—Figure 4f and Figure S5a, spermine—Figure 4g), HBSL1, a regulator of fetal hemoglobin levels (Stadhouders et al., 2014) recently associated with end‐of‐storage RBC ATP (Nemkov, Stephenson, et al., 2024) (spermidine—Figure 4f and Figure S4b), solute carrier SLC45A4 (spermidine—Figure 4f, *N*‐acetylspermidine, putrescine—Figure S4c), and translation initiator CTIF (spermidine—Figure S5a). SNPs on SMOX (spermine oxidase) were associated with spermine and *N*‐acetylspermidine levels (Figure 4g and Figure S5b). Finally, spermine levels associated with SNPs on glutaredoxin (GLRX), IZKF1, and hexokinase 1 (HK1, Figure 4g, Figure S5c). In brief, these analyses not only inform on the existence of common polymorphisms in humans that impact RBC metabolism in genetic regions encoding established rate‐limiting steps of arginine metabolism, but also identify novel regulatory nodes that can inform future strategies for pharmacologically manipulating this pathway. Despite the REDS cohort being exclusively constituted of volunteers who are sufficiently healthy to donate blood, some of the novel metabolite‐gene associations we identified herein involve multiple missense mutations (non‐synonymous coding or nonsense decay mutations—Table S1), including TRIM58 rs3811444 (associated with putrescine and L‐aspartate levels), G6PD rs1050828 (African variant coding for V68M, associated with spermidine), GCDH rs1799918 (associated with ornithine), or USP8 rs3743044 (associated with creatine).

### 3.4 Impact of genetic ancestry on RBC arginine metabolism

As donor ethnicity impacted arginine metabolism across donors of different chronological ages, we performed another mQTL analysis of REDS index multi‐omics data as a function of genetic ancestry. Genetic ancestry data were available for 13,029 (of the 13,091) REDS index donors of African, Asian, Hispanic, or European descent, the latter group accounting for >50% of the total donor population (Page et al., 2021). To summarize the results, arginine metabolites were plotted as a network and connected by edges to nodes, representative of genes, if they mapped to that coding region: results show more SNP–metabolite associations in donors of European descent, who were numerous and hence statistically powered to appreciate genome‐wide adjuster significant associations (*n* = 7943), and also in donors of African descent (*n* = 1503), in contrast to donors of Asian and Hispanic descent (*n* = 1560 and 986, respectively—Figure S6a,b; Table S1). The mQTL analyses identified three sets of associations: (i) associations unique across ancestries; (ii) associations consistent across ancestries; and (iii) associations in only the most numerous population (European). Unique associations across ancestries were seen (Figure S6c), including spermidine with TRIM58 and HBS1L in donors of European descent, and with G6PD only in donors of African descent. Similarly for creatine, the G6PD SNP association was significant for African‐descended donors, while a separate set of SNPs emerged as highly significant for altering RBC creatine levels in donors of European descent, linking it to SLC43A3, stomatin (STOM), IZKF1, and glycophorin E (GYPE—Figure S6c). In contrast, the association of spermine levels with SNPs on genes encoding SMOX and GLRX were consistently significant across all four ancestry groups (Figure S6d). Finally, leveraging ancestry‐specific mQTL analyses to characterize SNP–metabolite associations in the full cohort were mostly driven by European donors, the most represented group (Figure S6e).

### 3.5 Correlates to arginine in fresh and stored RBCs from diversity outbred mice

**FIGURE 5:**
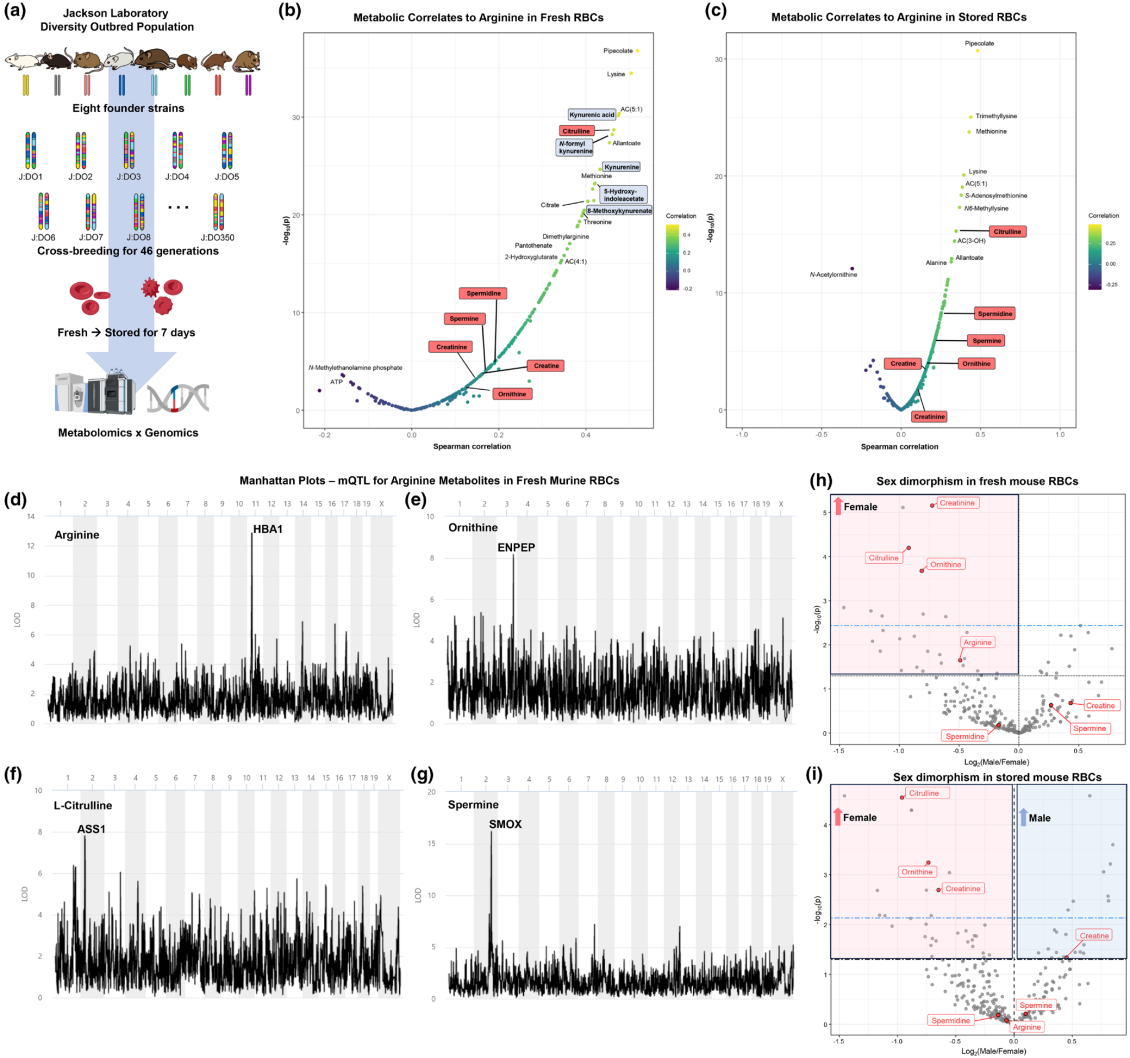
Arginine metabolism in fresh and stored murine RBCs (*n* = 525 animals). (a) Experimental overview of the generation of diversity outbred mice whose RBCs were characterized by multi‐omics approaches at Days 0 and 7 of storage. (b and c) Metabolite correlations (Spearman) to arginine levels in fresh (b) and stored (c) RBCs. Arginine pathway metabolites are highlighted in red. (d–g) Manhattan plots of metabolite–SNP associations in fresh RBCs from mice, highlighting arginine (d), ornithine (e), citrulline (f), and spermine (g). (h and i) Volcano plot illustrating the effects of sex on levels of arginine pathway metabolites (shown in red) in fresh (h) and stored (i) mouse RBCs. Blue shaded region indicates arginine metabolites increased in RBCs from males versus females; light red shaded region indicates arginine metabolites increased in RBCs from females versus males.

The REDS Index study was to samples obtained at end‐of‐storage. To disambiguate the impact of storage and genetics on RBC heterogeneity in arginine metabolism, we leveraged the Jackson Laboratory Diversity Outbred (DO) mouse population (Churchill et al., 2012), obtained by outbreeding eight founder strains for >50 generations (Nemkov, Key, Stephenson, et al., 2024). Metabolomics analyses were performed on fresh and 7‐day stored RBCs from these mice (*n* = 525) (Howie et al., 2019) (Figure 5a). This allowed studies of RBCs in a tractable system and a controlled environment (e.g., conditions that minimize effects of diet or other exposures on RBC metabolism); nonetheless, all mice were of comparable ages, allowing analysis only on the basis of sex and genetics. As an internal validation of omics data quality, we first correlated arginine levels in fresh (Figure 5b) and stored RBCs (Figure 5c) to the whole metabolome; these results included multiple positively correlated metabolites in the arginine pathway, including citrulline, ornithine, creatine/creatinine, and polyamines. Interestingly, ornithine's positive correlation with arginine was stronger in fresh than in stored RBCs. Notably, tryptophan catabolites also strongly correlated with arginine levels in stored RBCs, evidenced by kynurenic acid, *N*‐formylkynurenine, kynurenine, 5‐hydroxyindoleacetate, and 8‐methyoxykynurenate; this is interesting given similar observations regarding arginine metabolism and kynurenine in the context of aging in human RBCs (Figures 1 and 2).

Metabolite–SNP associations in the DO mice were determined by mQTL analysis for arginine metabolites (Table S1), which substantiated several observations in the large REDS cohort, including arginine with HBA1 (Figure 5d), ornithine with ENPEP (glutamyl aminopeptidase—Figure 5e), citrulline with ASS1 (Figure 5f,g), and spermine with SMOX (spermine oxidase Figure 5g). Regarding sex, creatinine, citrulline, ornithine, and arginine are elevated in RBCs from female versus male mice both fresh and after 7d storage whereas creatine is slightly elevated in male RBCs (vs. female) after storage (Figure 5h,i). Several of these findings are opposite of the sex dimorphism observed in humans at end‐of‐storage (Figure 3a), specifically creatinine and arginine, suggesting storage‐specific sexual dimorphic effects on this pathway warranting further investigation in the recalled donor cohort containing longitudinal storage data.

### 3.6 Arginine metabolism is associated with vesiculation and hemolysis in vitro and in vivo

**FIGURE 6:**
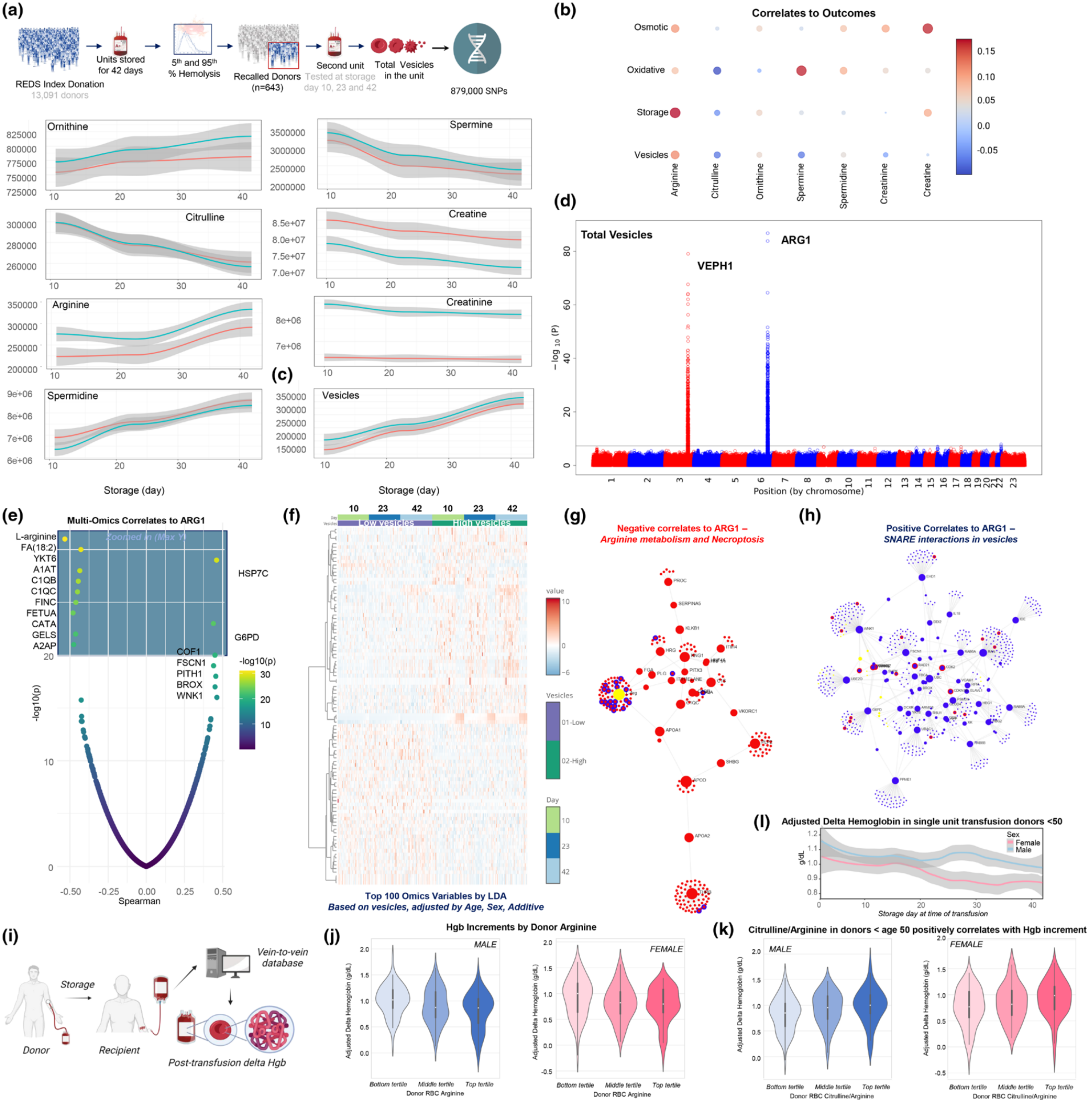
RBC arginine metabolism is associated with vesiculation and hemoglobin increments. (a) Vesicles were counted and single nucleotide polymorphisms (SNPs) were monitored in the REDS recall study for units from extreme hemolyzers (*n* = 643) sampled at Days 10, 23, and 42 of storage. Relative levels of arginine metabolites and vesicle counts are presented as a function of sex during the storage timecourse. Line colors: light blue for RBCs from males, light red for RBCs from females. (b) Spearman correlations of arginine pathway metabolites to storage duration and RBC functional outcomes (vesiculation, osmotic hemolysis, and oxidative hemolysis). Node color represents correlation coefficient (R); node size represents p‐value (larger node is smaller *p*‐value). (c) Vesicle counts as a function of donor sex and storage duration. (d) Manhattan plot of QTL associations with vesiculation implicates SNPs on VEPH1 and ARG1 with end‐of‐storage vesicle counts. (e) Spearman correlation of ARG1 multi‐omics correlates. (f) Heat map of top 100 features by linear discriminant analysis (LDA) of REDS recall cohort multi‐omics data at storage Days 10, 23, and 42 for the highest and lowest vesicle count units at each time point (*n* = 50 per vesiculation group). G‐H) Network analyses of top negative (i) and positive (j) correlates to ARG1. (i) Overview of vein‐to‐vein database for hemoglobin increments. (j) Adjusted delta hemoglobin levels (g/dL) at 24 h post single unit transfusion versus donor RBC arginine tertiles. Data for male donors are shown in blue, data for female donors are shown in pink. (k) Adjusted delta hemoglobin levels (g/dL) at 24 h post single unit transfusion versus donor RBC citrulline to arginine ratio tertiles for donors younger than age 50 and end‐of‐storage units. Data for male donors are shown in blue, data for female donors are shown in pink. (l) Adjusted delta hemoglobin levels (g/dL) at 24 h post single unit transfusion versus unit storage duration as a function of sex for donors younger than age 50.

Arginine metabolites, the top markers of donor chronological age, were examined as a function of RBC storage age (Figure 6a) in the REDS recall cohort. This identified storage‐associated decreases in citrulline and spermine levels and increases in arginine, ornithine, and spermidine (Figure 6a). Sex dimorphism was evident for arginine and creatinine, with higher levels in males, and creatine, with higher levels in females (Figure 6a, Table S1).

To understand the translational relevance of these changes, we correlated the end‐of‐storage levels of arginine metabolites with functional markers of RBC aging in vitro, including the propensity to hemolyze spontaneously (i.e., “storage hemolysis”) or following oxidant or osmotic stress (Figure 6b). This analysis identified strong positive correlations between arginine levels and all hemolysis parameters, creatinine levels and osmotic hemolysis, and polyamine levels and oxidative hemolysis (Figure 6b). In particular, arginine was the top positive correlate to the degree of vesiculation (Figure 6b). Total vesicle counts increased linearly during refrigerated storage (Figure 6c), suggesting that it is a suitable proxy for the unit storage age. When using vesicle counts as a quantitative trait for QTL analysis, total vesicle counts in a unit at the end‐of‐storage were associated with ARG1 coding region polymorphisms (Figure 6d, Figure S5d), the same region associated with arginine levels (Figure 4e), and with ventricular zone expressed PH domain containing 1 (VEPH1) (Figure 6d, Figure S5d), a gene implicated in TGFβ signaling. To pursue this observation, we correlated all omics data to ARG1 protein levels in all units from the recall donor cohort: top negative correlates include its substrate arginine, along with linoleic acid (FA 18:2), SNARE protein YKT6 (Kriegenburg et al., 2019), complement system proteins C1QB and C1QC, fibronectin, and catalase, a hydrogen peroxide‐scavenging enzyme (Figure 6e). Heat shock protein HSP7C and G6PD emerged as top positive correlates with ARG1, the latter underscoring the connection between RBC arginine metabolism and NADPH generation.

Linear discriminant analysis (LDA) of multi‐omics data from REDS recall donor units at storage Days 10, 23, and 42 showed clustering based on vesicle counts upon ranking of donors based on the highest and lowest vesicle counts at each time point (*n* = 50 per group—Figure 6f). Network analyses of the top pathways based on the variables informing sample clustering along LD1 indicated that elevated ARG1 protein levels in stored RBCs were associated with arginine metabolism and protection from necroptosis, whereas a positive association with ARG1 correlates with vesiculation as a function of SNARE interactions, which facilitate vesicle transport and dynamics (Figure 6g,h).

Vesiculation is a hallmark of RBC aging in vitro and in vivo, as vesicle shedding removes damaged components, decreasing RBC volume and deformability thereby increasing the likelihood of splenic sequestration for RBCs <43 μm^2^ (Roussel et al., 2021) (Figure 6a). Given the strong association between arginine/ARG1 and vesiculation, we hypothesized that arginine metabolite levels in donor RBCs would also associate with post‐transfusion hemolysis and transfusion efficacy in vivo, the latter measured by hemoglobin increments in recipients of single unit transfusions. To this end, we leveraged the REDS (index) vein‐to‐vein database, linking donor biology parameters to recipients' transfusion outcomes, with a specific focus on arginine and citrulline, the top positive correlate of donor chronological age (Figure 6i). After stratifying donor units by arginine and citrulline:arginine ratios into tertiles, we identified significant associations between levels of these metabolites and lower or higher hemoglobin increments, respectively (Figure 6j,k, Table S1). Given the impact of sex dimorphism and donor chronological age on arginine and citrulline levels in stored RBCs, we found these effects were most significant with donors younger than 50 and with units stored longer than 35 days (highest vesiculation group—Figure 6l). Taken together, these data amplify the relevance of modulations of RBC arginine metabolism in vivo with human aging by also implicating this pathway both in ex vivo RBC aging in blood banks and in transfusion efficacy of long‐stored units. Thus, this suggests that transfusing units with higher arginine levels (and, thus, more vesiculation) will yield smaller hemoglobin increments when transfused near their outdate. These data also indicate the relevance of modulations of RBC arginine metabolism in vivo by human aging and implicate this pathway in ex vivo RBC aging in blood banks and in transfusion effectiveness of long‐stored units.

## 4 DISCUSSION

Metabolic dysregulation and mitochondrial dysfunction are well‐established hallmarks of aging (López‐Otín et al., 2023). As such, most aging studies on metabolism have focused on characterizing biofluids (e.g., plasma (Darst et al., 2019; Johnson et al., 2019; Tian et al., 2023; Yao et al., 2024)) and mitochondria‐containing cells (Castro et al., 2022), especially immune cells and highly metabolically active organs whose metabolic dysfunction contributes to age‐related comorbidities (Hackett et al., 2023), such as brain (Ding et al., 2021; Jové et al., 2014), muscles (Cavalli et al., 2017), liver (Porukala & Vinod, 2023), heart (Rizza et al., 2014), kidneys (Jiao et al., 2022), and adipose tissue (Mancini et al., 2021). In contrast, limited literature exists on metabolic impacts of aging on cells without mitochondria, like RBCs (Domingo‐Ortí et al., 2021). While multiple studies examined aging of circulating RBCs (D'Alessandro et al., 2013; Jamshidi et al., 2020; Magnani et al., 1983), the present study focuses on the impact of organismal aging on RBC metabolism.

This study leverages RBCs from four cohorts of healthy individuals and SCD patients to characterize age‐dependent changes in arginine metabolism. These cohorts comprise >15,700 specimens from 13,757 adults, thus >70‐fold larger than prior studies. Multi‐omics findings from the largest cohort, >13,000 healthy American adults, were integrated with mQTL analyses and a vein‐to‐vein database to observe how changes in donor biology affect arginine metabolism and transfusion outcomes. Of note, three cohorts included older individuals healthy enough to donate blood, potentially introducing a “survival” or “healthy donor” bias in our sample pool (Atsma et al., 2011). Nonetheless, confirming similar findings in SCD patients, where these metabolites were previously associated with cardiorenal dysfunction, overall hazard ratio, and mortality (D'Alessandro, Anastasiadi, Tzounakas, Nemkov, Reisz, Kriebardis, Zimring, Spitalnik, & Busch, 2023; D'Alessandro, Nouraie, Zhang, Cendali, Gamboni, Reisz, Zhang, Bartsch, Galbraith, Espinosa, et al., 2023), mitigates some of these concerns. In addition, as the mQTL analyses were performed by linking genotypes to metabolite measurements in stored human blood products, we also validated our findings by testing both fresh and stored RBCs from 350 diversity outbred mice.

Because they lack mitochondria, RBC metabolism can mirror systems‐wide, non‐cell autonomous dysregulation. For example, correlating subject age and RBC levels of the CoA precursor pantothenate may reflect systemic availability, thereby indirectly informing on fatty acid oxidation capacity. In addition, pantothenate levels, combined with intra‐RBC fatty acids and acylcarnitine pools, may reflect enhanced membrane lipid damage repair via the Lands cycle in RBCs approaching the end of their lifespan (Nemkov, Key, Stephenson, et al., 2024). Most metabolomics studies on organismal aging to date identified a role for impaired fatty acid oxidation and cofactor homeostasis—especially the depletion of NAD pools (Covarrubias et al., 2021)—as etiological contributors to age‐related metabolic dysfunction and associated “inflammaging” (Cannizzo et al., 2011; Minhas et al., 2019). An integrated multiorgan analysis of aging in mice recently reported increases in immune proteins across all tissues, consistent with a global pattern of immune infiltration (Keele et al., 2023). Dysregulated NAD synthesis from impaired tryptophan metabolism (Yaku et al., 2018)—in both at the microbiome (Montgomery et al., 2020) and host—was linked to depleted tryptophan pools and accelerated aging phenotypes (e.g., progeria and Down syndrome (Powers et al., 2019)). This dysregulation is partly explained by NAD(P) breakdown by nucleotidases like CD38 (Covarrubias et al., 2021; Nemkov et al., 2023), and by pathologically activating the cGAS‐STING (Gulen et al., 2023)‐interferon (Powers et al., 2019)‐dependent kynurenine pathway, a cascade that can be activated by detecting endogenous mitochondrial nucleic acids as foreign (Zecchini et al., 2023), consistent with the endosymbiotic theory of mitochondrial incorporation into eukaryotic cells (Murphy & O'Neill, 2024). Notably, we confirmed (Nemkov, Stephenson, Erickson, et al., 2024) that RBC kynurenine levels rank among the top correlates of aging across all four cohorts investigated in this study. Importantly, a relay pathway was identified between tryptophan and arginine metabolism, which regulates the inflammatory state of innate immune cell types that become dysfunctional with aging (Mondanelli et al., 2017; Moss et al., 2023). The observed associations herein among RBC levels of arginine and tryptophan metabolites with organismal age were observed both in human and murine RBCs, fresh and stored, further substantiating a likely cross‐regulation of these pathways.

Several RBC arginine metabolites ranked among the top 10 correlates to the chronological age of the subject across all four cohorts. Arginine, a conditionally essential amino acid, is the substrate for multiple enzymes critical for cell growth and survival. In particular, arginine metabolism by nitric oxide synthase (NOS) yields citrulline and nitric oxide (NO), the latter relevant for vasodilation, inflammatory signaling, and oxidative modification of biomolecules. Although trace levels of functional NOS were described in RBCs (Leo et al., 2021), their role in the context of RBC or organismal aging remained unexplored. However, in the current study, citrulline, a NOS product and top positive correlate with donor chronological age, was most strongly associated with a CPS1 SNP previously linked to persistent neonatal pulmonary hypertension (El‐Khazragy et al., 2021). NOS requires NADPH; notably, our mQTL analysis identified previously unappreciated linkages between associated metabolites (e.g., putrescine, spermidine, and *N*‐acetylspermidine) and a polymorphic region on chromosome X coding for G6PD, the rate‐limiting enzyme of NADPH generation via the pentose phosphate pathway in RBCs. Approximately 500 million people worldwide carry variant G6PD alleles (Luzzatto et al., 2020); the most frequent (e.g., the “common” Mediterranean variant and the African variant), destabilize the enzyme, impairing NADPH generation, glutathione function, and redox homeostasis (Francis et al., 2020). Another notable linkage between glutathione systems and arginine metabolism is inferred by the observed association between spermine levels and glutaredoxin, an enzyme that reduces glutathionylated proteins at reactive cysteine residues.

Arginine is metabolized by ARG1 to form ornithine and urea as the final step of the urea cycle, which is incomplete in mature RBCs devoid of mitochondria. Further metabolism of ornithine yields either polyamines or creatine and agmatine. While RBCs do not harbor the rate‐limiting enzyme of the polyamine pathway, ornithine decarboxylase, they do express cationic solute carriers (e.g., SNPs on SLC45A4—present on RBCs (Haiman et al., 2024)—were significantly associated with spermidine levels in donors of European and Caucasian descent); the carriers participate in transport of polyamines between bloodstream compartments, thus indirectly modulating their bioavailability for other processes, such as immunomodulation (McCubbrey et al., 2022).

Polyamines, particularly spermidine, are very interesting because of their roles in immunomodulation (McCubbrey et al., 2022), age‐related hematopoiesis (Kumar et al., 2023), and lifespan extension (e.g., in yeast, flies, and worms) (Eisenberg et al., 2009). Spermidine is upstream of histone acetylation and mediates autophagy and protein translation in B cells (Eisenberg et al., 2016) and T cells (Alsaleh et al., 2020), thereby protecting against age‐related degeneration and disease progression (Soda, 2022). The spermidine precursor putrescine declined in the RBCs and plasma of healthy Caucasian volunteers (*n* = 193) starting around age 60 (Sanchez et al., 2023). Age‐associated decreases in human RBC spermine (*n* = 117 subjects) were suggested previously though the findings did not reach statistical significance and no clear trend for spermidine was observed (Elworthy & Hitchcock, 1989). Similarly, spermine decreased with age in post‐mortem human liver although spermidine levels did not correlate with age (Uemura et al., 2020). Our data are consistent with these studies: RBC spermine decreased almost linearly with subject chronological age, although results for spermidine are less clear, decreasing with subjects' age in REDS and Walk‐PHaSST, increasing with age in DIDS. In the aforementioned study of aging in post‐mortem human liver, expression of spermine oxidase SMOX, which oxidizes spermidine to spermine, increased with age, while expression of ornithine decarboxylase (which catalyzes ornithine to putrescine) decreased (Uemura et al., 2020). SMOX coding region polymorphisms were herein linked to RBC spermine levels in J:DO mice, as well as in human donors across all ancestries. In RBCs, spermine adversely affects intracellular calcium concentration, which impairs membrane integrity (Kucherenko & Lang, 2010).

A pathway‐level understanding of arginine biochemistry throughout aging has not yet been clearly defined. Arginine is a critical hub of nitrogen metabolism in cellular and tissue function, and limited evidence suggests it is deregulated in RBCs during organismal aging. An NMR metabolomics study of a small cohort of healthy donors found citrulline and pantothenate as top metabolites in RBCs from older versus younger individuals (*n* = 15 for each) (Chaleckis et al., 2016). Moreover, Percoll gradient separation of older circulating RBCs from younger ones showed lower arginine levels in older RBCs (Bizjak et al., 2015). In this view, it is worth noting that our studies focused on bulk RBC sub‐populations, and some of the observations reported herein may be influenced by a heterogeneous distribution of RBCs of different ages across donors, in keeping with what we reported for young and old donors as a function of biological sex (Mykhailova et al., 2020). Arginine metabolism as a function of RBC sub‐population in circulation remains incompletely documented (D'Alessandro et al., 2013; Jamshidi et al., 2020), though it is known that some transporters are lost from RBC membranes as they age in circulation, such as in the case of creatinine (Ku & Passow, 1980).

Relevant beyond transfusion medicine, ARG1 is an immune system regulator, and arginine supplementation of mice of various ages boosted immune responses (Lewis & Langkamp‐Henken, 2000). We found that the ARG1 coding region (and RBC levels of the corresponding protein) was polymorphic in humans. These polymorphisms were associated with arginine levels and storage vesicle counts, the latter a hallmark of RBC aging in vivo and ex vivo (D'Alessandro, Nouraie, Zhang, Cendali, Gamboni, Reisz, Zhang, Bartsch, Galbraith, Gordeuk, & Gladwin, 2023) as shedding membrane‐derived vesicles alters RBC rheology, increasing splenic retention and extravascular hemolysis (Roussel et al., 2021). Refrigerated storage exacerbated changes to RBC arginine metabolism independent of donor age, particularly in longer stored units. To explore translational implications, we leveraged a vein‐to‐vein database, discovering that high‐arginine units yielded lower post‐transfusion hemoglobin increments, while higher citrulline units (normalized to arginine) were associated with higher increments. ARG1 is responsible for ornithine generation and is a critical regulator—via competition for the same substrate—of RBC‐NOS, thereby indirectly regulating NO export (Yang et al., 2013). However, it remains to be conclusively determined how ARG1 SNPs affect enzymatic activity and steady‐state arginine metabolite pools, and ultimately the mechanistic links between altered arginine metabolism in donors and the full picture of transfusion efficacy in recipients of their donated RBC units. As such, while our findings inform on the impact of donor chronological age and biological (e.g., sex and BMI) or genetic factors on inter‐donor heterogeneity in arginine metabolism, vesiculation, and hemolytic propensity, this information could be leveraged to inform blood inventory management strategies beyond the standard first‐in‐first‐out approach, whereby units from certain categories of donors (e.g., older, carrying SNPs relevant to arginine metabolism, with higher levels of certain arginine metabolites at donation) are preferentially issued on a priority basis. Independently from informing novel precision transfusion medicine strategies, the present findings also expand our understanding of arginine metabolism as a function of biological and genetic factors, which is relevant to aging and age‐related comorbidities in which anomalies in this pathway have been causally implicated and/or identified as potential therapeutic targets (Gad, 2010; He et al., 2024; Hofer et al., 2022; Madeo et al., 2018; Minois et al., 2011; Morselli et al., 2009; Nam et al., 2024; Polis et al., 2021; Viltard et al., 2019; Wang et al., 2020; Xu et al., 2020).

## AUTHOR CONTRIBUTIONS

JAR wrote the manuscript which was critically reviewed and approved by all authors. JAR, TN, DS, AK, and AD performed method development, quality control, and metabolomics data acquisition and analysis. MTG designed and supervised the Walk‐PHaSST study; EAH and SLS designed and supervised the DIDS study; EJE, GRK, and GPP performed mQTL analyses; NR performed analysis via the vein‐to‐vein database; MD and KCH performed proteomics studies; EJE, GRK, GPP, and AD performed statistical analyses; PJN, SK, and MPB performed and supervised the REDS RBC Omics study; EJE, AD, JAR, and AK prepared figures. AD and JAR revised the paper, which was critically reviewed and finalized by all co‐authors.

## CONFLICT OF INTEREST STATEMENT

The authors declare that AD, KCH, and TN are founders of Omix Technologies Inc. and Altis Biosciences LLC. AD and SLS are Scientific Advisory Board (SAB) members for Hemanext Inc. AD is a SAB member for Macopharma Inc. Dr. Gladwin is a co‐inventor of patents and patent applications directed to the use of recombinant neuroglobin and heme‐based molecules as antidotes for CO poisoning, which have been licensed by Globin Solutions, Inc. Dr. Gladwin is a shareholder, advisor, and director in Globin Solutions, Inc. Dr. Gladwin is also co‐inventor on patents directed to the use of nitrite salts in cardiovascular diseases, which were previously licensed to United Therapeutics, and now licensed to Globin Solutions and Hope Pharmaceuticals. Dr. Gladwin is an inventor on an unlicensed patent application directed at the use of nitrite for halogen gas poisoning and smoke inhalation. Dr. Gladwin was a principal investigator in a research collaboration with Bayer Pharmaceuticals to evaluate riociguate as a treatment for patients with SCD, which has concluded. Dr. Gladwin is a textbook author and receives royalties from MedMaster Inc., and is a textbook editor and receives royalties from McGraw‐Hill. All the other authors have no conflicts to disclose in relation to this study.

## Supporting information


Appendix S1.

Appendix S2.

## Data Availability

The mouse data and code to produce the reported results are available at https://doi.org/10.6084/m9.figshare.24456619. Processed data and results can also be viewed, explored, and downloaded using the QTLViewer (Vincent et al., 2022) webtool (https://churchilllab.jax.org/qtlviewer/Zimring/RBC). Omics data for additional analysis is available in the online supplement; requests for additional data may be made to the corresponding author at angelo.dalessandro@cuanschutz.edu.
