# Supplementary material for: Arginine metabolism is a biomarker of red blood cell and human aging
Source: Aging Cell. 2024 Oct 30;24(2):e14388. doi: 10.1111/acel.14388 (PMC11822668; doi:10.1111/acel.14388)
Supplement: Supplementary file 1 — Appendix S1. [file ACEL-24-e14388-s001.pdf]

SUPPLEMENTARY MATERIAL

Arginine metabolism is a biomarker of red blood cell and human aging

Julie A. Reisz,[1] Eric J. Earley,[2] Travis Nemkov,[1,3] Alicia Key, [1] Daniel Stephenson,[1] Gregory R. Keele,[2] Monika Dzieciatkowska,[1] Steven L. Spitalnik,[4] Eldad A. Hod,[4] Steven Kleinman,[5] Nareg H. Roubinian,[8] Mark T. Gladwin,[6] Kirk C. Hansen,[1,3] Philip J. Norris, [7,8] Michael P. Busch,[7,8] James C. Zimring,[9] Gary A. Churchill,[10] Grier P. Page,[2] Angelo D'Alessandro[1,3*]

1) Department of Biochemistry and Molecular Genetics, University of Colorado Anschutz Medical Campus, Aurora, CO, USA;
2) RTI International, Atlanta, GA, USA;
3) Omix Technologies Inc, Aurora, CO, USA;
4) Department of Pathology and Cell Biology, Columbia University Irving Medical Center, New York, NY, USA;
5) University of British Columbia, Victoria, British Columbia, Canada;
6) University of Maryland School of Medicine, University of Maryland, Baltimore, MD, USA;
7) Vitalant Research Institute, San Francisco CA, USA;
8) Department of Laboratory Medicine, University of California San Francisco, CA, USA;
9) Department of Pathology, University of Virginia, Charlottesville, VA, USA;
10) Jackson Laboratories, Bar Harbor, ME, USA

***Corresponding author:**

Angelo D'Alessandro, PhD

Department of Biochemistry and Molecular Genetics

University of Colorado Anschutz Medical Campus

12801 East 17th Ave., Aurora, CO 80045

Phone # 303-724-0096

E-mail: angelo.dalessandro@cuanschutz.edu

TABLE OF CONTENTS

Supplementary Figures 2

SUPPLEMENTARY FIGURE 1 2
SUPPLEMENTARY FIGURE 2 3
SUPPLEMENTARY FIGURE 3 4
SUPPLEMENTARY FIGURE 4 5
SUPPLEMENTARY FIGURE 5 6
SUPPLEMENTARY FIGURE 6 7

SUPPLEMENTARY FIGURES

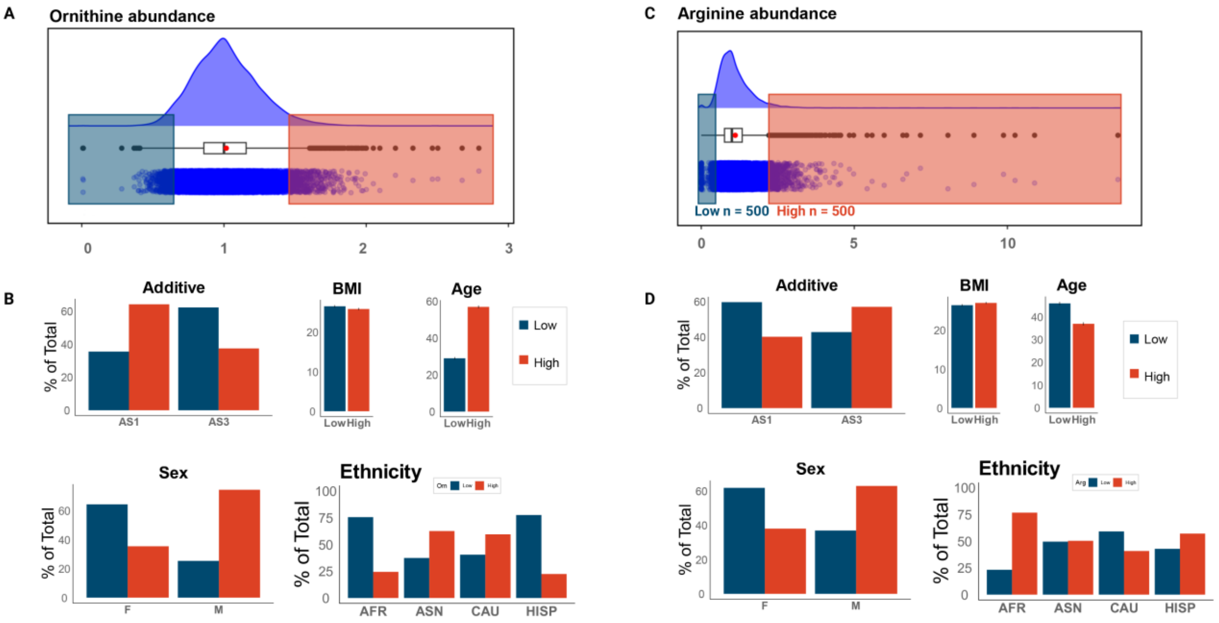

Figure S1. A) Raincloud plot of RBC ornithine levels highlighting the top and bottom 500 donors. B) Representation among the donors with top and bottom 500 end-of-storage ornithine levels across storage additive, BMI, age, sex, and ethnicity, each shown as a percentage of total for the 1000 donor subset identified in (A). C) Raincloud plot of RBC arginine levels highlighting the top and bottom 500 donors. D) Representation among the donors with top and bottom 500 end-of-storage arginine levels across storage additive, BMI, age, sex, and ethnicity, each shown as a percentage of total for the 1000 donor subset identified in (C). AFR = African American, ASN = Asian, CAU = Caucasian, HISP = Hispanic. Data is presented as median +/- SEM.

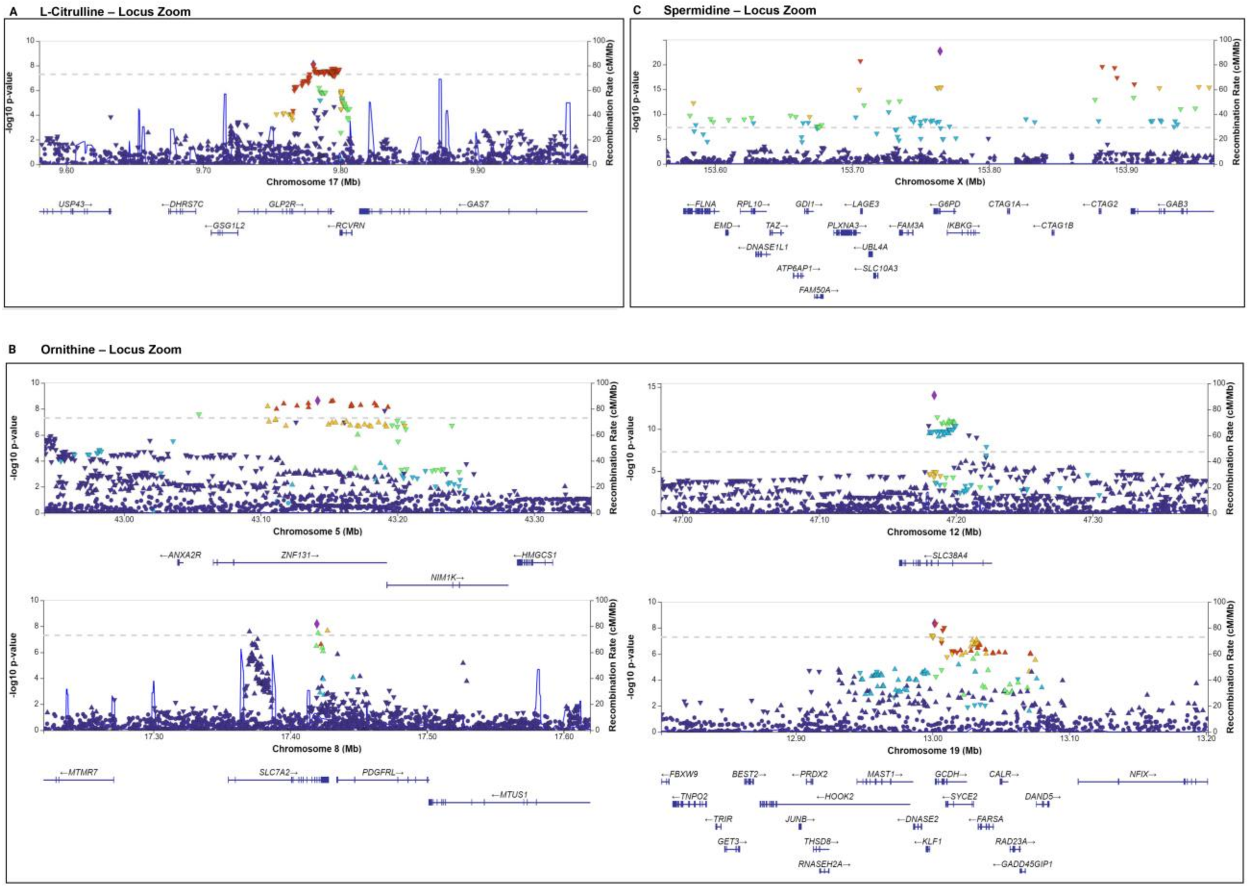

Figure S2. A) Locus Zoom supporting the association of citrulline with polymorphic GLP2R. B) Locus Zoom for the associations of ornithine with polymorphic regions of the genome encoding for ZNF131, SLC7A2, SLC38A4, and GCDH. C) Locus Zoom for the association of spermidine with G6PD on chromosome X.

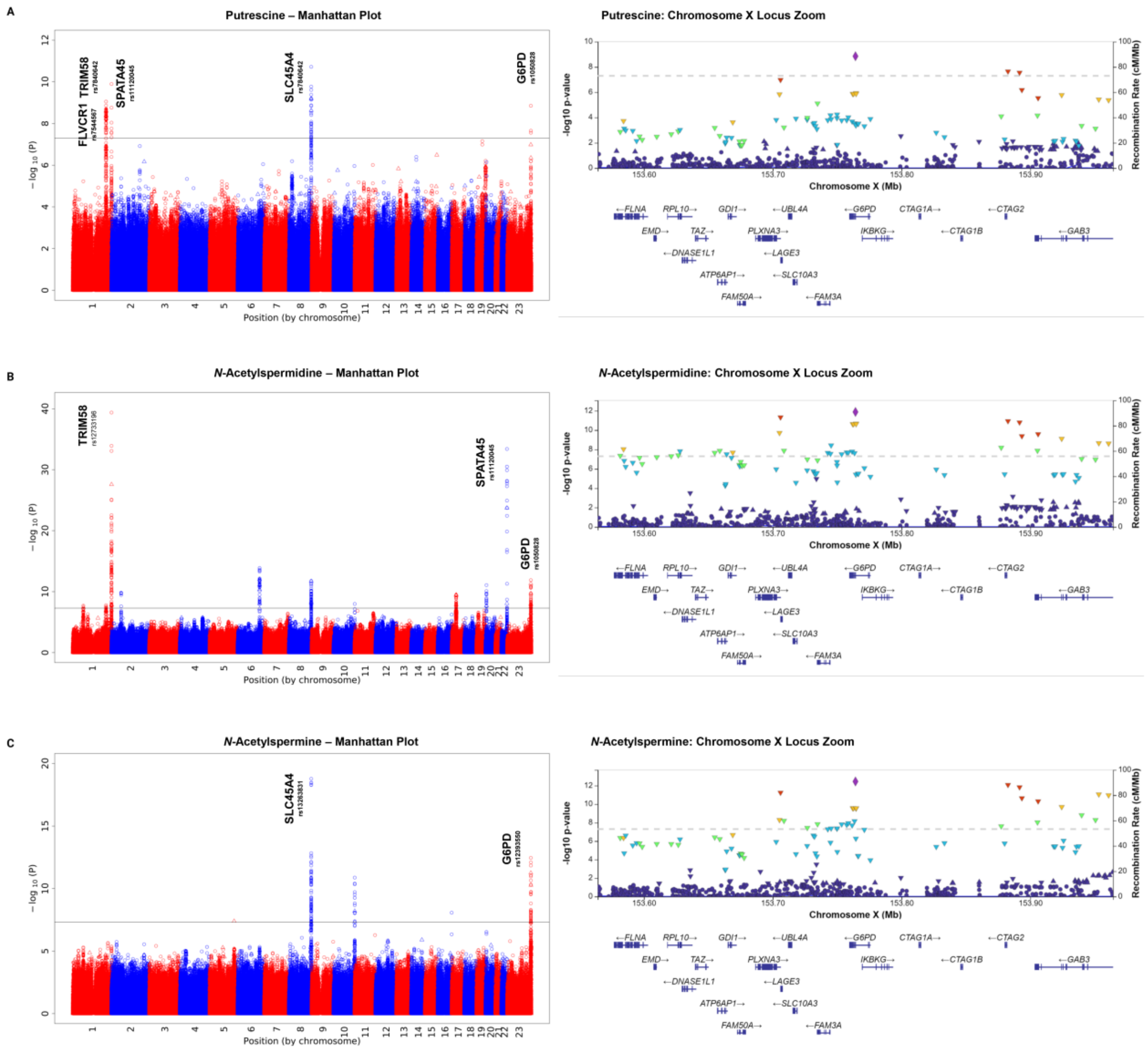

Figure S3. A) Manhattan plot illustrating top putrescine-SNP associations (left) and Locus Zoom of the polymorphic region corresponding to G6PD (right). B) Manhattan plot illustrating top *N*-acetylspermidine-SNP associations (left) and Locus Zoom of the polymorphic region corresponding to G6PD (right). C) Manhattan plot illustrating top *N*-acetylspermine-SNP associations (left) and Locus Zoom of the polymorphic region corresponding to G6PD (right).

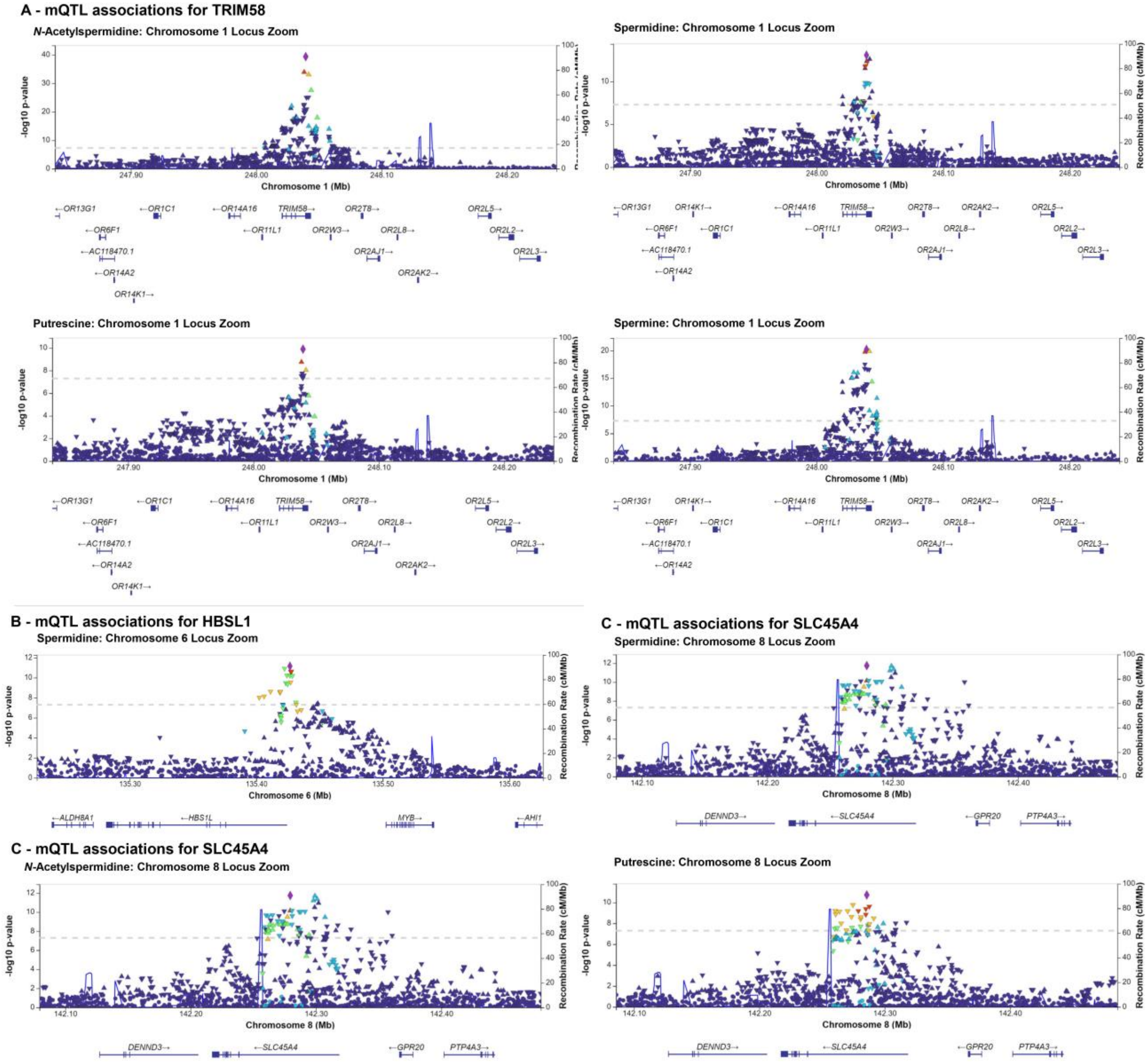

Figure S4. A) mQTL associations with polymorphic TRIM58. B) mQTL associations with polymorphic HBSL1. C) mQTL associations with polymorphic SLC45A4.

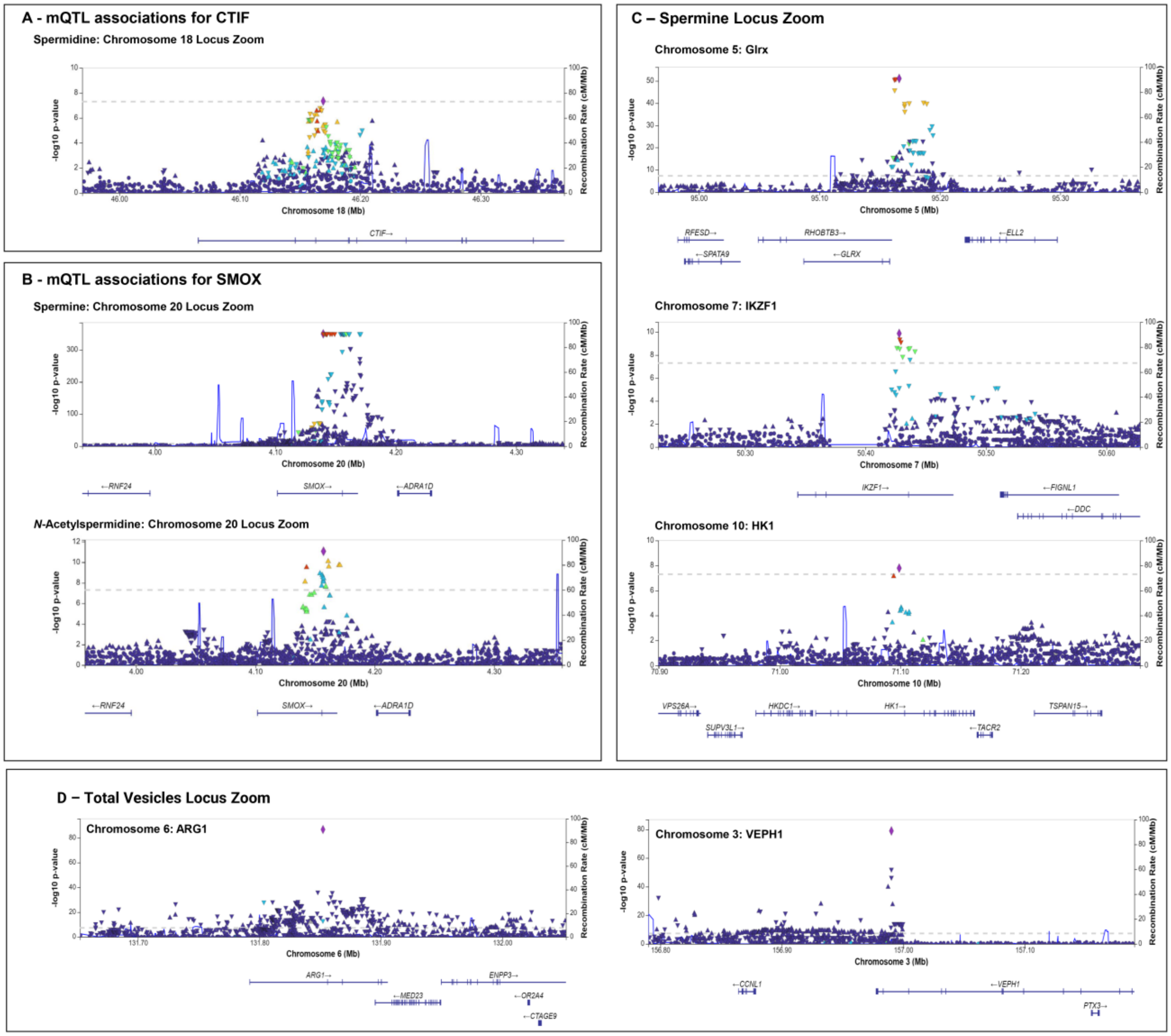

Figure S5. A) mQTL associations with polymorphic CTIF. B) mQTL associations with polymorphic SMOX. C) Locus Zoom plots for associations of spermine with glutaredoxin (Glrx1), IKZF1, and hexokinase 1 (HK1). D) Locus Zoom plots for the QTL association of vesicle counts with ARG1 on chromosome 6 and VEPH1 on chromosome 3.

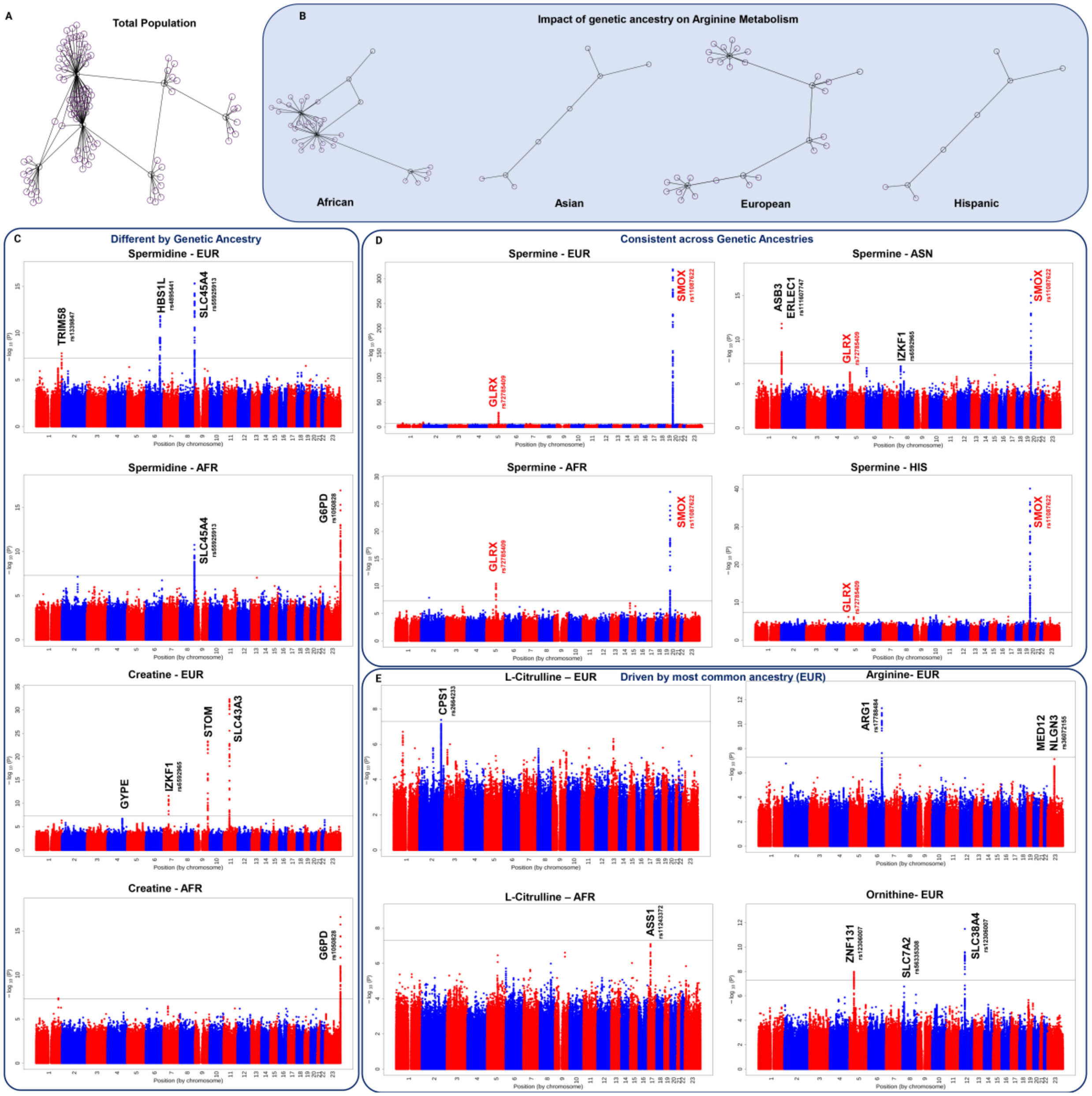

Figure S6. Metabolite Quantitative Trait Loci (mQTL) analysis by genetic ancestry reveals shared and unique metabolite-SNP associations across ancestry groups. A) Network analysis of metabolite-SNP associations in the total REDS index population (n = 13,029 donors for which ancestry was available). B) Network analysis of metabolite-SNP associations for each ancestry by continent. C) Manhattan plots showing unique associations by genetic ancestry. D) Manhattan plots of metabolite-SNP associations common across ancestries. E) Manhattan plots of identified metabolite-SNP associations driven by European ancestry, the most highly represented ethnicity group in the REDS index cohort.
